# Prediction of Protein−compound Binding Energies from Known Activity Data: Docking‐score‐based Method and its Applications

**DOI:** 10.1002/minf.201700120

**Published:** 2018-02-14

**Authors:** Yoshifumi Fukunishi, Yasunobu Yamashita, Tadaaki Mashimo, Haruki Nakamura

**Affiliations:** ^1^ Molecular Profiling Research Center for Drug Discovery (molprof) National Institute of Advanced Industrial Science and Technology (AIST) 2-3-26 Aomi, Koto-ku, Tokyo 135-0064 Japan; ^2^ Technology Research Association for Next-Generation Natural Products Chemistry 2-3-26, Aomi, Koto-ku Tokyo 135-0064 Japan; ^3^ IMSBIO Co., Ltd. Owl Tower, 4-21-1 Higashi-Ikebukuro, Toshima-ku Tokyo 170-0013 Japan; ^4^ Institute for Protein Research Osaka University 3-2 Yamadaoka Suita, Osaka 565-0871 Japan

**Keywords:** Binding free energy, ChEMBL, Docking score, Protein−compound docking

## Abstract

We used protein−compound docking simulations to develop a structure‐based quantitative structure−activity relationship (QSAR) model. The prediction model used docking scores as descriptors. The binding free energy was approximated by a weighted average of docking scores for multiple proteins. This approximation was based on a pharmacophore model of receptor pockets and compounds. The weights of the docking scores were restricted to small values to avoid unrealistic weights by a regularization term. Additional outlier elimination improved the results. We applied this method to two groups of targets. The first target was the kinase family. The cross‐validation results of 107 kinase proteins showed that the RMSE of predicted binding free energies was 1.1 kcal/mol. The second target was the matrix metalloproteinase (MMP) family, which has been difficult for docking programs. MMPs require metal‐binding groups in their inhibitor structures in many cases. A quantum effect contributes to the metal−ligand interaction. Despite this difficulty, the present method worked well for the MMPs. This method showed that the RMSE of predicted binding free energies was 1.1 kcal/mol. In comparison, with the original docking method the RMSE was 1.7 kcal/mol. The results suggest that the present QSAR model should be applied to general target proteins.

## 1 Introduction

The quantitative structure−activity relationship (QSAR) approach is a useful tool for optimizing leads and predicting target/off‐target activities and toxicity. QSAR‐based affinity predictions are useful for the general drug development process, including the repositioning (repurposing) of already approved drugs, poly‐pharmacology, and the prediction of drug−drug interactions.^1^, ^2^, ^3^, ^4^, ^5^, ^6^, ^7^, ^8^, ^9^, ^10^, ^11^, ^12^, ^13^, ^14^, ^15^ The recent accumulation of protein−compound affinity data in public repositories, such as the PubChem and ChEMBL projects, has enabled us to carry out proteome‐wide target/off‐target predictions.^16^,^17^ These predictions are based on QSAR models for multiple proteins, just as in conventional computer‐aided drug design and virtual screening.

Wide application of QSAR‐based models in computer‐aided drug development, such as protein−compound binding free energy (affinity) prediction, target/off‐target predictions, and counter screening based on QSAR models, has succeeded in many studies, including ours.^3^,^4^,^7^,^8^ Most QSAR models rely on descriptors with sets of two‐dimensional (2D) substructures; the most popular such descriptors are MDL's MACCS key and 0‐3D molecular descriptors (e.g., 5,270 descriptors recorded in Dragon (Kode srl, Pisa, Italy)). In our previous studies, we developed QSAR methods for the affinity prediction of a compound by using docking studies against multiple proteins.^17^, ^18^, ^19^ We used a protein−compound affinity matrix as the set of descriptors and applied principal component regression (PCR).^18^ The ***Q*** ^***2***^ value of calculated binding free energies was 0.44 and the RMSE was 1.54 kcal/mol for about 97 kinases and 18,491 compounds selected from the ChEMBL database. However, the coefficients of the regression equations for some targets were unrealistically (10^3^–10^5^) higher than those for other targets. Either these coefficients should be restricted to a range of realistic values, or the applicability domain should be very restricted around the known experimental data.

In the present study, we applied a combination of the ridge (Tikhonov regularization) regression, robust estimation, and principal component analysis to the protein−compound affinity matrix.^19^‐^21^ The robust estimation was expected to reduce the problem of error in the experimental data. The present method restricted the coefficients of the generated prediction equation around realistic values. The method was applied to the kinases and the matrix metalloproteinases (MMPs) of the ChEMBL database.^22^, ^23^, ^24^

MMPs, which have zinc ions in the reaction pockets, require metal‐binding groups in their inhibitor structures in many cases.^25^ The metal−ligand interaction shows quantum effects such as electron donation and back donation, that form a weak covalent bond, in addition to the electrostatic and van der Waals interactions.^26^, ^27^, ^28^, ^29^, ^30^, ^31^, ^32^, ^33^, ^34^, ^35^, ^36^ The quantum effect makes it difficult to estimate binding energy. A method to evaluate metal interaction has long been sought. In the framework of the classical force field, several methods have represented metal interactions. One is to add a metal contact term such as the van der Waals−type potential and the potential considering the coordination number of the central metal ion.^27^, ^28^, ^29^, ^30^, ^31^ The other method is to modify the parameters of the atomic charge and the van der Waals potential of the original force field.^32^, ^33^, ^34^ The metal parameters depend on the environments of the metal atom, so the user should tune the parameters for each protein.^35^,^36^ The metal contact terms enable the user to reproduce protein−ligand complex structures and the absolute value of the binding energy. However, protein‐dependent parameter tuning has been a time‐consuming process, especially when the user analyzes multiple target proteins.

In addition, we evaluated the effect of the elimination of outlier data points from these multiple data points corresponding to each single protein−compound pair, and we improved the present regression model. The method, which we call the “docking‐score‐based QSAR model”, predicted the protein−compound binding affinities of 107 kinases that have no metals in their pocket, and those of 5 MMPs (MMP2, MMP3, MMP7, MMP9, and MMP13) that have a zinc ion in each pocket. The docking‐score QSAR method worked for these various targets. Namely, the RMSE values were ∼1 kcal/mol, respectively.

## 2 Materials and Methods

### 2.1 Background of Prediction Models

In the present study, we develop a binding‐energy (affinity) prediction method based on the protein−compound docking scores obtained by a docking program; the present method is a modified version of our QSAR method.^18^

In our present and previous QSAR models, the affinity of compounds can be estimated by using a pharmacophore model of the target protein. The IUPAC guidelines define a pharmacophore as “an ensemble of steric and electronic features that is necessary to ensure the optimal supramolecular interactions with a specific biological target and to trigger (or block) its biological response”.^37^ Hydrogen donors, hydrogen acceptors, hydrophobic groups of ligands and receptors are called “pharmacophore features” and the functional groups can give these features.^38^ These features are usually depicted as spheres connected by lines those lengths represent the spatial distances among these features of the ligands and receptors. In the present study, we temporarily define a pharmacophore as a set of spatially distributed pharmacophore features. Each pharmacophore feature represents the probability of existence of a hydrogen‐bond donor, a hydrogen‐bond acceptor, and both electrostatic and hydrophobic interaction sites. Both receptors and ligands have pharmacophores. We approximate that the receptor−ligand binding energy is given by the sum of interactions between the pairs of the ligand pharmacophore (***φ***_***l***_) and the receptor pharmacophore (***φ***_***r***_). Here, we introduce a function for the interaction ***k*** and let ***k***(***φ***_***r***_, ***φ***_***l***_) be an interaction value between the two pharmacophores.

Because we discuss only the interaction, we do not need to explicitly represent ***φ***s, but rather we need only the value of the function ***k***(***φ***_***r***_, ***φ***_***l***_) for the interaction. Our final ΔG‐estimation equation does not include ***φ***s explicitly but consists of docking scores. In this framework, the interaction between the receptor and ligand pharmacophores gives the binding energy of the pharmacophore *l* in a ligand to the pharmacophore *r* in a protein, ***Δ*** ***G**_l_* ^*r*^, as

(1) $$\Delta G_{l}^{r}=g_{l}^{r}k\left(\phi_{r},\phi_{l}\right)$$

where *g_l_* ^*r*^ is a parameter.

We try to generalize this discussion by introducing a linear combination of a basic pharmacophore. Suppose ***Φ*** (= {***φ***_***1***_, ***φ***_***2***_, ***φ***_***3***_, …..}) is a set of all pharmacophores. The ***φ*** functions form the basis set of the pharmacophores of any kind of ligand or receptor. Each pharmacophore (***φ***_***i***_) does not have to be found in an actual protein structures. In this discussion, pharmacophores work as descriptors of both protein and compound. The pharmacophores are used only to derive a regression model, and we do not calculate them explicitly. And the total number of pharmacophores could be infinite in the present discussion. We suppose that the binding energy of the i‐th compound and the *r*‐th pharmacophore in a protein is

(2) $$\Delta G_{i}^{r}=\sum_{l\in i}g_{l}^{r}k\left(\phi_{r},\phi_{l}\right)=\sum_{l\in i}\Delta G_{l}^{r},$$

**Figure 1:**
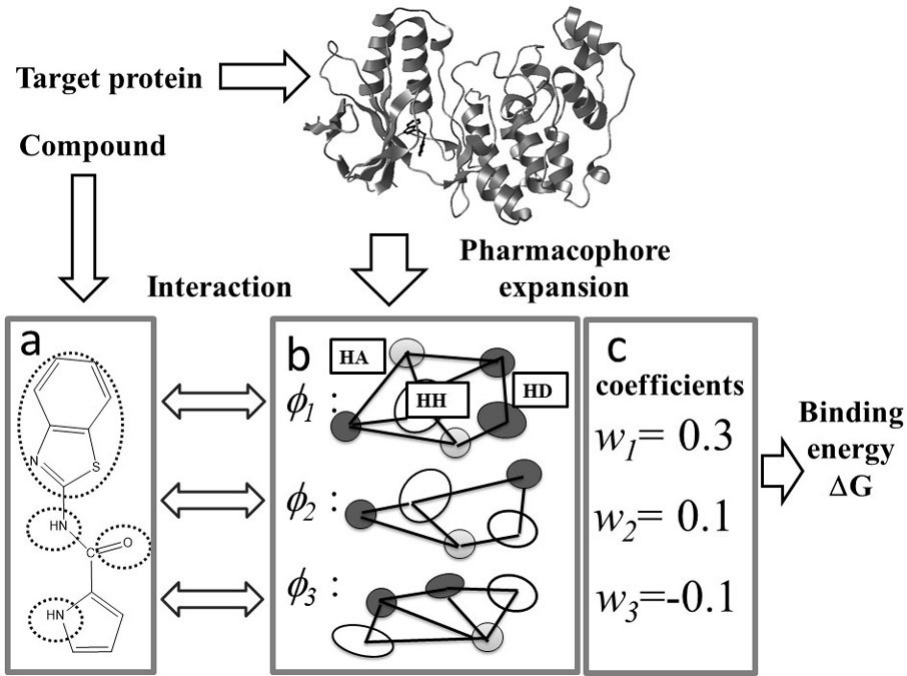
Schematic representation of the pharmacophore expansion of a ligand‐binding site of a protein. The interaction between the ligand and protein pharmacophores gives the binding energy. **a**: Example of pharmacophore of the ligand pharmacophore. The dotted circles represent the pharmacophore features. **b**: Example of pharmacophores. HH, HA, and HD indicate hydrophobic, hydrogen‐bond acceptor, and hydrogen‐bond donor sites, shown by open, grey and black circles, respectively. Only three pharmacophores (φ_1_, φ_2_, and φ_3_) are depicted. The lines represent the specific distances between pharmacophore features of each pharmacophore. **c**: Example of pharmacophore expansion of the receptor pharmacophore following eqs. 2 and 3.

Then the protein−compound binding energy (***Δ*** ***G***_***i***_^***a***^) between the a‐th protein and the i‐th compound is given by the following linear combination of binding energies to pharmacophores {***φ***_m_; m=1, 2, 3,…}, where *w_r_* ^*a*^ are the scaler coefficients (see Figure 1).

(3) $$\Delta G_{i}^{a}=\sum_{r\in a}w_{r}^{a}k\left(\sum_{l\in i}g_{l}^{r}k\left(\phi_{r},\phi_{l}\right)\right)=\sum_{r\in a}w_{r}^{a}\Delta G_{i}^{r}$$

Various protein pockets correspond to the various pharmacophores, and they work as probes for a given compound instead of ***φ*** in eqs. 2 and 3. Thus the binding free energy can be estimated by the regression based on the docking scores for the various protein structures. This is a simplified model, since it does not include the intramolecular interaction or the conformational entropy of the compound.

**Figure 2:**
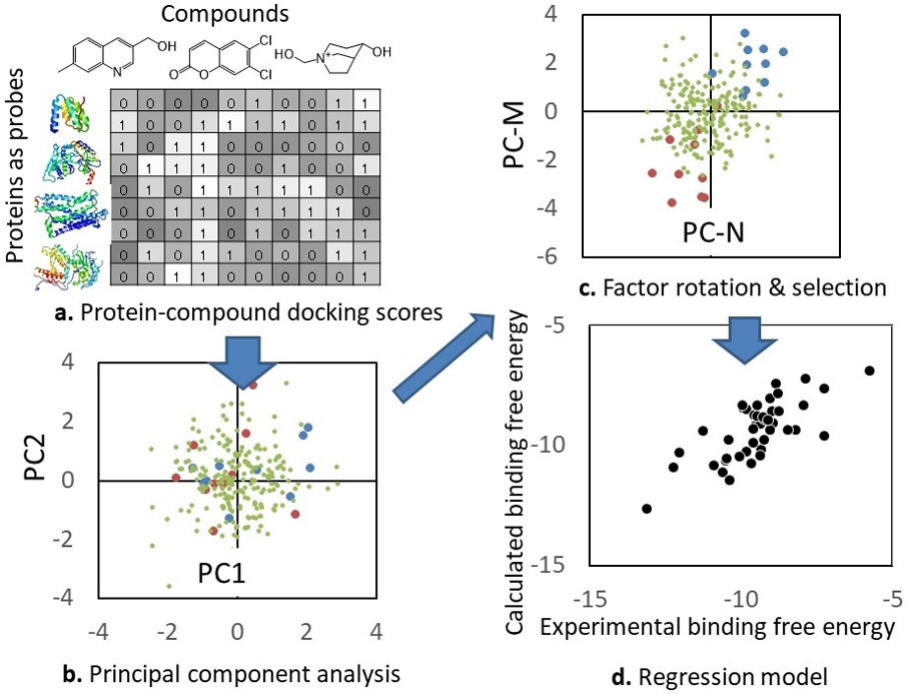
Schematic representation of the docking‐score QSAR method. **a**: The table represents a protein−compound docking score matrix. In the table, the values are depicted in grayscale. **b**: The PC analysis projects the score vector (column vector) of each compound into a point in the PC space. In the PC analysis, each dot represents a compound. The red, green, and blue dots represent the strong, medium, and weak affinity compounds, respectively. In this example, the first and second principal component axes (PC1 and PC2) are not useful to describe the affinity difference. **c**: The factor rotation method selected the representative axes (PC‐M and PC‐N) from the total N_p_ axes. PC‐N and PC‐M describe the affinity difference clearly. **d**: Finally, the regression model is constructed by using PC‐N and PC‐M.

Figure 2 shows the procedure of the present QSAR method. This method requires a learning set of 3D structures of compounds, the binding energy data between those compounds, and target proteins. We assume that protein−ligand docking programs give ***Δ*** ***G***_***i***_^***a***^. We proposed an approximation, as follows.^18^

(4) $$\Delta G_{i}^{a}\approx \sum_{b}R_{b}^{a}\cdot s_{i}^{b}+\beta^{a}$$

Here, ***s***_***i***_^***b***^, ***R***_***b***_^***a***^, and ***β*** ^***a***^ are the docking score of the *i*‐th compound to the *b*‐th protein, the weight parameter, and the parameter for fitting, respectively. The set of {***b***} can include the target protein (the *a*‐th protein). Equation 4 showed the RMSE of predicted binding energies was 1.5 kcal/mol.^18^ One of the most serious problems with QSAR models is, in general, the limited range of applicability domains, since these models cannot work for input data that is too different from the training data set.^39^ Since docking scores have been developed to mimic binding free energy, we assume that a docking score is equal to the binding free energy, ***Δ*** ***G***_***i***_^***a***^ *=**s***_***i***_^***a***^, ***R***_***a***_^***a***^=1, ***β*** ^***a***^=0, and ***R***_***b***_^***a***^=0 for ***a***≠***b*** in eq. 4. In this case, eq. 4 can work without any experimental affinity data, and the problem of identifying an applicability domain is avoided.

Eq. 4 gives a linear regression model whose descriptors are docking scores, and the number of parameters is equal to the number of proteins.

In the previous^18^ and present models, the protein (*a*) – compound (*i*) binding energy ***Δ*** ***G***_***i***_^***a***^ is approximated by the PCR method based on the protein−compound docking scores {***s***_***i***_^***b***^}. The optimal principal component (PC) axis was selected to maximize the correlation coefficient by the leave‐one‐out (LOO) cross‐validation test. The PC axes are selected by factor rotation (see Figure 2). The factor rotation method selects the axes that show major contributions in the PC analysis among the total axes. We rewrote eq. 4, as follows.

(5) $$\Delta G_{i}^{a}=\sum_{j=1}^{Naxis}c_{j}^{a}\cdot p_{i}^{j}+\beta^{a}$$

(6) $$p_{i}^{j}=\sum_{b=1}^{Np}d_{b}^{j}\cdot \left(s_{i}^{b}-\bar{s^{b}}\right).$$

Here, ***c***_***j***_^***a***^, ***β*** ^***a***^, ***p***, and ***d***_***b***_^***j***^ are the parameter, offset parameter, principal component vector, and loading vector, respectively. The total number of optimal PC axes is N_axis_. The upper bar represents an average. The PC axis of the protein−compound docking‐score matrix ***s*** gives the loading vector ***d*** and the principal component vector (axis) ***p***. The parameters ***c*** and ***β*** are determined by multilinear regression (MLR).

*N_axis_* and *N_p_* are the number of selected axes by the factor rotation and the total number of proteins used in the docking study as the compound descriptors, respectively. Here, *N_axis_* (*N_axis_*<*N_p_*) is determined to maximize the correlation coefficient obtained by the LOO cross‐validations. The parameters are determined based on the learning set and then are used for prediction.

To calculate the protein−compound docking scores ***s***_***i***_^***b***^, we used our own program, Sievgene,^40^,^41^ a protein−ligand flexible docking program for *in silico* drug screening. Sievgene is a part of the myPresto system, which is available online (http://presto.protein.osaka‐u.ac.jp/myPresto4/) and is free for academic use.

### 2.2 New Prediction Model with Restricted Regression Method

In the present study, we introduced a regularization term and robust estimation into the previously developed QSAR model based on docking scores using eqs. 5–6. In eq. 5, the coefficients ***c***_***j***_^***a***^ and ***β*** were determined by the multiple linear regression in our previous study.^18^ In some cases, ***c***_***j***_^***a***^ was unrealistically large (10^3^–10^5^). The value should be 0 or 1 when the target protein structure is used in the docking calculation (we call this ***c***_***j***_^***a***^ value “ideal value”), and the docking score is equal to the binding energy. We would like to restrain ***c***_***j***_^***a***^ to around this values. In the present study, the coefficients were determined by minimizing the objective function by introducing the regularization term. Let *Objf (a)* be the objective function for the *a‐*th target protein for the determined parameters ***c*** and ***β***.

(7) $$Obif\left(a\right)=\sum_{i=1}^{N_{cmp}}{\left(\Delta G_{\exp_{i}}^{a}-\left(\sum_{j=1}^{Naxis}c_{j}^{a}\cdot p_{i}^{j}+\beta^{a}\right)\right)}^{2}+\lambda \sum_{j=1}^{Naxis}{\left(c_{j}^{a}-c_{ideal_{j}}^{a}\right)}^{2}$$

Here, ***c***_***ideal j***_^***a***^ is the ideal value of ***c***_***j***_^***a***^, and ***β*** ^***a***^ and ***λ*** are parameters. *N_cmp_* is the total number of compounds. ***c***_***ideal j***_^***a***^ is unknown. ***Δ*** ***G***_***exp***_ represents the experimental ***Δ*** ***G*** value. The last term restrains ***c***_***j***_^***a***^ to around the ideal values. Equation 7 is a generalized version of the Tikhonov regularization.^19^, ^20^, ^21^ To estimate the ***λ*** value, we considered the following things. Since eq. 4 suggest that *c_i_* ^*a*^=***Δ*** ***G***_***i***_^***a***^ under the ideal conditions (***Δ*** ***G***_***i***_^***a***^ *=**s***_***i***_^***a***^, ***R***_***a***_^***a***^=1, ***β*** ^***a***^=0, and ***R***_***b***_^***a***^=0 for a≠b, discussed in section 2.1) and that {***c***_***j***_^***a***^} values should correspond to ***Δ*** ***G*** values. The larger the ***λ*** value is, the smaller the {***|c***_***j***_^***a***^|} values are. In general, most |***Δ*** ***G***|<18 kcal/mol. In the present study, ***λ*** was set to 0, 00001, 0.0002,.., 0.01, to satisfy {***|c***_***j***_^***a***^|}<18 kcal/mol. Also, ***c***_***ideal j***_^***a***^ was set to 0, since the protein set providing the docking scores does not include the target protein structures in the present study.

In addition, we apply the maximum‐likelihood‐like estimation (M estimation), a robust estimation method.^42^ The M estimation method weights the difference between a calculated value and an experimental value considering the predicted experimental error. The M estimation version of our objective function is given as follows

(8) $$Obif\left(a\right)=\;\sum_{i=1}^{N_{cmp}}w\left(d_{a}^{i}\right){\left(\Delta G_{\exp_{i}}^{a}-\left(\sum_{j=1}^{Naxis}c_{j}^{a}\cdot p_{i}^{j}+\beta^{a}\right)\right)}^{2}+\lambda \sum_{j=1}^{Naxis}c_{j}^{a^{2}}$$

where

(9) $$w\left(d\right)=\left\{\begin{array}{c} {\left(1-{\left(\frac{d}{W}\right)}^{2}\right)}^{2}\;\left|d\right|\leq W\\ 0\;\left|d\right|>W \end{array}\right.$$

and

(10) $$d=\sum_{i=1}^{N_{cmp}}\Delta G_{\exp_{i}}^{a}-\left(\sum_{j=1}^{Naxis}c_{j}^{a}\cdot p_{i}^{j}+\beta^{a}\right)$$

Here, ***W*** and ***d*** are the upper limit of allowed error and a scalar value, respectively. The ***d*** value is the difference between the experimental value and the fitted value. The parameters ***c*** and ***β*** are determined to minimize the objective function. The derivation of eq. 8 is not linear, and we solve eqs. 8–10 by an iterative procedure. The M estimation method places a higher weight on likely reliable data than unreliable data. ***W***=0, 5, 10, 15,…, 100 were examined in the present study. We call this model (eq. 8) the “docking score QSAR model”.

### 2.3 Generation of the Docking‐score Index by Protein−compound Docking

The protein−compound docking scores ***s***_***i***_^***b***^ were calculated by the protein−compound docking program Sievgene.^40^ This ligand‐flexible program reconstructed about 50% of the receptor−compound complexes in PDB (132 in total) with an accuracy of less than 2 Å root mean square deviation (RMSD) in a self‐docking test.^40^ The computational setup in the present study was exactly the same as that in the previous study. Namely, Sievgene generated up to 100 conformers for each compound, and 200×200×200 grid potentials were adapted for all proteins. The pocket regions were suggested by the coordinates of the original ligands in the receptor−compound complex structures, and each edge length of the grid was about 35–45 Å. The docking‐scoring function is based on the physical chemistry (accessible surface area, van der Waals potential, and electrostatic potential). The estimated error in binding free energy is almost 2.5 kcal/mol.^42^ It takes 1 second to dock one compound against one protein on a single core of a Xeon 5570 CPU (2.98 GHz).

### 2.4 Probe Protein Sets

To generate {***s***_***i***_^***b***^ **}** in eqs. 5–8, we performed a protein–compound docking simulation based on the soluble protein structures registered in the Protein Data Bank (PDB). The probe protein set consisted of 600 arbitrarily selected protein structures, as in our previous study (see APPENDIX A in Supporting Information). All of these structures were protein−ligand complexes. The protein set did not include the present target proteins. For protein sets, the complexes containing a covalent bond between the protein and ligand were removed, and all missing hydrogen atoms were added to form all‐atom models of the proteins. All water molecules and cofactors were removed from the protein structures. All Asp and Glu were prepared as negatively charged forms, while Lys and Arg were prepared as positively charged forms. The atomic charges of the proteins were the same as those in AMBER parm99.^43^ The docking pocket of each protein was indicated by the coordinates of the original ligand.

### 2.5 Training Set: Target Proteins and Compounds

To compare the present and previous results, the compounds and their assay information (compound structures, affinities against kinases) were downloaded from the Kinase SARfari website (https://www.ebi.ac.uk/chembl/sarfari/kinasesarfari/downloads) in the ChEMBL database, as in our previous work.^18^ Note that the ChEMBL main page does not link to the KinaseSARfari website directly. The biochemical assay data, namely, *K_i_*, *IC_50_*, %residual activity, and/or %inhibition values of human kinase protein‐inhibitor systems, were also extracted from the bioactivity table in KinaseSARfari, and these data were converted to binding free energy by the software package used in our previous report.^18^ The biochemical assay data were translated into the binding free energy by the Cheng−Prusoff equation and others.^14^,^44^. We assumed that the experimental conditions were the same in all the assays. The procedure is described in details in APPENDIX B in the Supporting Information.

The first target was the kinase family. As target proteins, 107 kinases were selected. The 3D structures of the compounds were energy‐optimized by cosgene^32^ with the general AMBER force field (GAFF),^45^ and the atomic charges were calculated by the MOPAC AM1 model using the Hgene program of the myPresto suite. Each functional group in all molecules was set to the dominant ionic form at pH 7. Finally, the filter condition reduced the number of data points used, and 45,663 assay data points of 107 kinases were derived.

The second target was the MMP family. We selected MMP2, MMP3, MMP7, MMP9, and MMP13. The protein structures were extracted from the PDB. The PDB IDs were 1hov, 2y6d, 4g9l, 5b5o, and 5cuh for MMP2, MMP3, MMP7, MMP9, and MMP13, respectively.^46^, ^47^, ^48^, ^49^, ^50^ The protein structures were prepared in the same manner as the kinases. The zinc atom charges of these MMPs were set to +2, which is the formal charge of the most stable singlet zinc ion. As mentioned in the previous works, the actual charges were smaller than the formal charge and the charge values should differ from each other depending on the pocket structures. The protein−compound interaction data were extracted from the ChEMBL database. The compound structures and the ΔG values were prepared in the same manner as the kinases.

### 2.6 Definitions of *Q* ^*2*^ and RMSE

The definition of ***Q*** ^***2***^ and root‐mean‐square error (RMSE) are determined as follows.

(11) $$Q^{2}=1-\frac{\sum_{i=1}^{N_{cmp}}{\left(\Delta G_{pred_{i}}-\Delta G_{\exp_{i}}\right)}^{2}}{\sum_{i=1}^{N_{cmp}}{\left(\Delta G_{\exp_{i}}-\bar{\Delta G_{\exp_{i}}}\right)}^{2}}$$

(12) $$RMSE=\sqrt{\frac{\sum_{i=1}^{N_{cmp}}{\left(\Delta G_{pred_{i}}-\Delta G_{\exp_{i}}\right)}^{2}}{N_{cmp}}}$$

Here, ***Δ*** ***G***_***pred***_, ***Δ*** ***G***_***exp***_, and the upper bar represent the predicted in validation and experimental ***Δ*** ***G*** values and the average, respectively. In the present study, we do not compare the ***Q*** ^***2***^ values of kinases to that of MMPs, since the variances of the experimental data were different to each other.^51^

## 3 Results and Discussion

### 3.1 Cross‐validation Tests of the Docking‐score QSAR Model

Each of the compounds that gave assay data for one or more of the 107 target proteins was docked to all proteins of a protein set to generate the protein−compound docking‐score matrix ***s***. Then we adopted eq. 8 with changes to ***λ*** and ***W*** values and the LOO cross‐validation test to calculate the ***Q*** ^***2***^. In addition, we applied the 4‐fold cross validation test in all kinase cases to verify the results.

Table 1 summarizes the RMSE and ***Q*** ^***2***^ values of ***Δ*** ***G***s calculated by the docking‐score QSAR model with changes to the ***λ*** value in eq. 8 without the M estimation. The ***Q*** ^***2***^ and RMSE depended on ***λ***, and ***λ***=0.0001 showed the best ***Q*** ^***2***^ and RMSE. These values did not change appreciably when ***λ*** was >0.00001 and <0.005. The regularization term worked well, and ***λ*** was set to around 0.0001–0.005 in the following calculations. In the present study, our regression model did not use the docking scores for the target proteins and instead used the 600 probe proteins. Indeed, we found the coefficient ***R***_***b***_^***a***^ values reasonably low. Namely, the maximum and minimum values of ***R***_***b***_^***a***^ were 3.9 and −3.8, respectively, and the average and standard deviations of ***R***_***b***_^***a***^ were 0.01 and 0.1, respectively.

**Table 1:** Average ***Q*** ^***2***^ and RMSE values obtained by the LOO cross validations of the docking‐score QSAR model with various *λ* and *W*=0 for all 107 proteins.

λ	Q^2^	RMSE (kcal/mol)	λ	Q^2^	RMSE (kcal/mol)
0	0.423	1.544	0.0005	0.704	1.074
0.00001	0.702	1.087	0.001	0.702	1.076
0.00002	0.703	1.081	0.002	0.687	1.089
0.00005	0.704	1.077	0.005	0.680	1.101
0.0001	0.705	1.074	0.01	0.669	1.122
0.0002	0.704	1.075	0.1	0.392	1.627

In eq. 8, the estimated error ***d*** was unknown a priori. The ***d*** value was estimated by an iterative solution method. Starting from ***d***=0, the new ***d*** value was estimated by using the previous ***d*** value. The iteration converged within 4–6 steps in all cases. Tables 2 and 3 summarize the ***Q*** ^***2***^, and RMSE of calculated ***Δ*** ***G***s obtained by the docking‐score QSAR model with changes to the ***W*** value in eq. 9. The data in Tables 2 and 3 were obtained by the LOO and 4‐fold cross validation tests, respectively. The ***Q*** ^***2***^ and RMSE values were improved when 20 kcal/mol<***W***<100 kcal/mol in many cases compared to the result by eq. 7 (***W*** value is “−“ in Tables 2 and 3) and the prediction with ***W***=5 kcal/mol gave the worst ***Q*** ^***2***^ and RMSE values (Figure S1). The ***Q*** ^***2***^ and RMSE values depended on ***W*** weakly, and the results with ***W***>20 kcal/mol were almost equal to each other. The M estimation improved the results slightly.

**Table 2:** Average ***Q*** ^***2***^ and RMSE values obtained by the LOO cross validations of the docking‐score QSAR model with various *W* for all 107 proteins.

W (kcal/mol)	λ=0.0001		λ=0.0002	
	Q^2^	RMSE (kcal/mol)	Q^2^	RMSE (kcal/mol)
–	0.702	1.087	0.704	1.075
5	0.622	1.27	0.623	1.268
10	0.698	1.099	0.698	1.097
15	0.704	1.077	0.704	1.078
20	0.704	1.076	0.704	1.075
50	0.705	1.074	0.705	1.074
100	0.704	1.076	0.705	1.074

**Table 3:** Average ***Q*** ^***2***^ and RMSE values obtained by the 4‐fold cross validations of the docking‐score QSAR model with various *W* for all 107 proteins.

W (kcal/mol)	λ=0.002		λ=0.005	
	Q^2^	RMSE (kcal/mol)	Q^2^	RMSE (kcal/mol)
–	0.629	1.236	0.629	1.225
5	0.577	1.345	0.579	1.334
10	0.627	1.240	0.627	1.230
15	0.629	1.235	0.629	1.225
20	0.628	1.236	0.629	1.224
50	0.628	1.236	0.629	1.224
100	0.629	1.235	0.629	1.224

**Figure 3:**
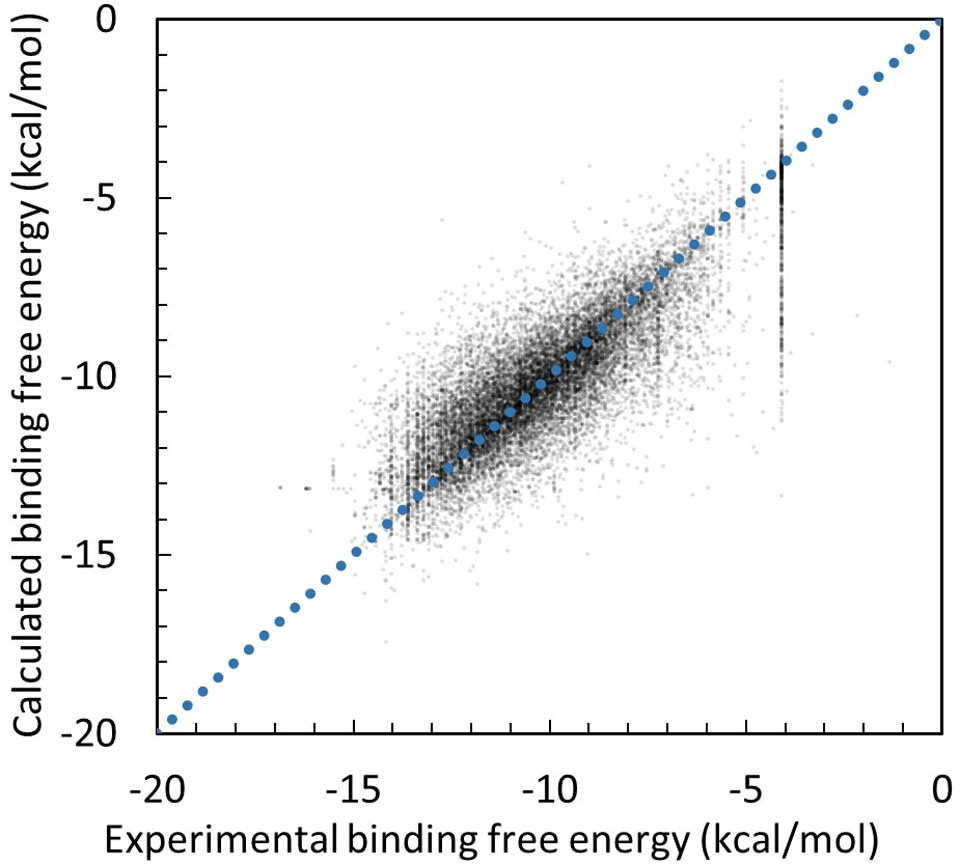
Correlation between the experimental and prediction data for all 107 kinase proteins obtained by the docking‐score QSAR model with ***λ***=0.0002 and ***W***=20 kcal/mol. The dots represent the predicted data points by the LOO cross‐validation test.

Equation 8 with ***λ***=0.0002 and ***W***=20 kcal/mol in the LOO cross validation and that with ***λ***=0.005 and ***W***=20 kcal/mol in the 4‐fold cross validation gave the best ***Q*** ^***2***^ and RMSE values. Figure 3 shows the results of the LOO cross‐validation test with ***λ***=0.0002 and ***W***=20 kcal/mol for all 107 kinases. The average error reached 1.08 kcal/mol and ***Q*** ^***2***^=0.70 in the binding free energy. Some of the datasets showed very high accuracy, considering that the thermal fluctuation was about 0.6 kcal/mol at room temperature (Table S1). The results of the 4‐fold cross‐validation test with ***λ***=0.005 and ***W***=20 kcal/mol for all 107 kinases showed qualitatively the same trends as those in Figure 3 (Table S2). The ***W*** value was originally in the acceptable error range, and the regression/analysis ignored the data points with the estimation error (***d***)>***W***. The optimal ***W*** value was much bigger than the standard deviation of ***Δ*** ***G*** values that are multiply observed for each target protein−compound pair (see section 3.2), meaning that the regression used all the data points with almost equal weight.

### 3.2 Effect of Elimination of Outliers from the Experimental Data

There are multiple experimental affinity data for some single protein−compound pairs in the database. This is because the experimental data depend on the experimental conditions such as pH, temperature, density of buffer salt, and cell line, and then the unique protein−compound pairs in the database correspond to these different experimental affinity data points under different experimental conditions. Some kinases are anti‐cancer drug targets, and the amino‐acid mutation of kinases causes drug resistance. The database includes such kinase data, and these mutants with different affinities share unique protein IDs in the database.

In this case, a drug‐resistant protein should show weaker binding affinity to the same compound than to a native protein. The enzyme activity could depend on the effector protein in the protein−protein interaction network. In this case, the observed enzyme activity depends on the experimental environment, such as whether it is *in vivo* or *in vitro*. Also, the protein activities depend on the temperature, pH, and density of the salt of the buffer solvent. These conditions are not shown in the ChEMBL database clearly.

The average standard deviation of the Log_10_ ***K***_***i***_ affinity data was about 0.24 kcal/mol, that of Log_10_ ***IC***_***50***_ was about 0.44 kcal/mol, and that of Log_10_ (%‐inhibition or %‐residual activity) was 0.093 kcal/mol. When all the experimental data were translated to ***Δ*** ***G***, the average deviation of the binding affinity was about 0.73 kcal/mol (Table S3).

We examine the effect of eliminating outliers among multiple data on the prediction result. When multiple affinity data correspond to a single pair of a protein ID and a compound ID, we eliminate outliers among the multiple data. Let the number of multiple data points for the *a*‐th protein and the *i*‐th compound be ***M***_***a***_^***i***^ and the *k*‐th affinity value be ***E***_***a***_^***i***^ *(k)*. We define ***E***_***a***_^***i***^ *(k)* as an outlier of the data set when ***E***_***a***_^***i***^ *(k)* satisfies the following relationship.

(13) $$\left|E_{a}^{i}\left(k\right)-\Delta G_{a}^{i}\right|>N\cdot \sigma_{a}^{i}$$

(14) $$\sigma_{a}^{i}=\sqrt{\frac{\sum_{m=1}^{M_{a}^{i}}{\left(E_{a}^{i}\left(m\right)-\Delta G_{a}^{i}\right)}^{2}}{M_{a}^{i}-1}}$$

where ***Δ*** ***G***_***a***_^***i***^ is the average value of a set of ***E***_***a***_^***i***^ *(m)*, *m*={1,.., *M_a_* ^*i*^}, and ***σ***_***a***_^***i***^ is the deviation of the data. Equation 14 defines the *k*‐th compound as an outlier when the *k*‐th compound satisfies this condition. In the present study, we removed outliers from all experimental data trying ***N***=0.2, 0.4, 0.5, 0.6, 0.8, 1, 2, and 3 before the following cross‐validation tests.

**Figure 4:**
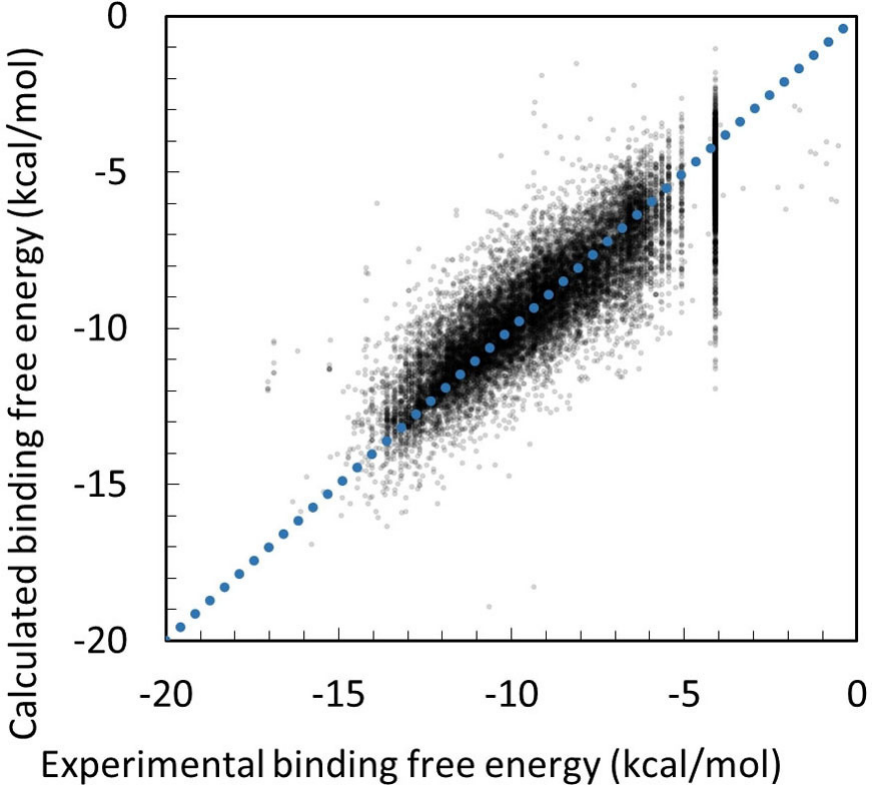
Correlation between experimental and prediction data for all 107 kinase proteins obtained by the docking‐score QSAR model eliminating outliers with λ=0.005, W=20 kcal/mol, and N=0.8. The dots represent the predicted data points by the 4‐fold cross‐validation test.

Table 4 summarizes the cross‐validation results for ***N***=0.2, 0.4, 0.5, 0.6, 0.8, 1, 2, and 3. In Table 4, the regression models used were eq. 8 with ***λ***=0.0002 (LOO cross validation) and ***λ***=0.005 (4‐fold cross validation), respectively. In both tables, ***W***=20 kcal/mol. In all cases, the elimination of outliers in the test data set improved the prediction results with decreasing ***N***. Elimination of outliers worked well when ***N σ*** was set to ***N***<0.8. Figure 4 shows the result of the 4‐fold cross‐validation test for all 107 kinases. The result by the LOO cross validation was similar to Figure 4. Some of the data points are located vertically at −4 kcal/mol. These data points correspond to 0% inhibitions or 100% residual activities. Except for these data points, the predicted data points clearly correlate to the experimental data. Also, we examined the 4‐fold cross validation case with ***λ***=0.002, and the result was close to that summarized in Table 5 (Table S4).

**Table 4:** Average ***Q*** ^***2***^ and RMSE values obtained by the LOO cross validation (*W*=20 kcal/mol and *λ*=0.0002) and 4‐fold cross validation (*W*=20 kcal/mol and *λ*=0.005) tests of the docking‐score QSAR model with various *N* for all 107 proteins.

Nσ	Total no. of compounds	LOO cross validation		4‐fold cross validation	
		Q^2^	RMSE (kcal/mol)	Q^2^	RMSE (kcal/mol)
0.2σ	35050	0.762	0.919	0.678	1.132
0.4σ	35736	0.763	0.913	0.680	1.134
0.5σ	36249	0.763	0.915	0.679	1.133
0.6σ	36804	0.758	0.918	0.678	1.136
0.8σ	38124	0.760	0.913	0.685	1.117
σ	44063	0.704	1.052	0.645	1.192
2σ	45549	0.694	1.079	0.631	1.220
3σ	45650	0.693	1.084	0.631	1.223

**Table 5:** ***Q*** ^***2***^ and RMSE data obtained by the Sievgene docking program and the LOO cross validation tests of the docking‐score QSAR model over all MMPs.

Protein	No of ligands	Naïve docking		Docking‐score QSAR	
		Q^2^	RMSE (kcal/mol)	Q^2^	RMSE (kcal/mol)
MMP‐2	489	0.004	1.81	0.774	1.23
MMP‐3	369	0.029	2.50	0.672	1.48
MMP‐7	98	0.058	0.99	0.922	0.46
MMP‐9	445	0.048	1.82	0.64	1.61
MMP‐13	148	0.044	1.34	0.903	0.62
Average	309.8	0.037	1.692	0.782	1.08

When ***N***=0.2, 0.5, 0.8, and 1.0, eqs. 13–14 eliminated 23%, 21%, 17%, and 4% of the data, respectively, out of the total of 45,663 data points. Since the standard deviation of the experimental ***Δ*** ***G*** values was 0.7 kcal/mol, the results of RMSE<0.7 kcal/mol should be the accurately predicted cases. The outlier elimination increased the accurately predicted cases (RMSE<0.7 kcal/mol) from 20 to 42 cases out of the 107 targets in the LOO cross validation.

We selected 24 targets arbitrarily from the total of 107 kinases examined above (Table S1) and analyzed the individual results carefully. For these targets, 12 protein structures were available in the PDB (Table S5). These protein coordinates were prepared in the same manner as described in sections 2.3 and 2.4. We applied the docking‐score QSAR method (***λ***=0.005, ***W***=20 kcal/mol, ***N***=0.8, 4‐fold cross validation) and the naïve docking. In each individual target, the docking‐score QSAR result was better than that by the naïve docking study (Figure S2). Namely, the average RMSE values by the naïve docking and the docking‐score QSAR were 1.6 and 1.2 kcal/mol, respectively. The average ***Q*** ^***2***^ values were 0.11 and 0.65 for the naïve docking and the docking‐score QSAR method, respectively (Table S5).

In some cases, the outlier elimination did not work and the prediction accuracy remained low. The data sets with more than 1000 data points showed particularly low accuracy, such as the sets for cyclin‐dependent kinase 2, epidermal growth factor receptor erbB1, tyrosine‐protein kinase SRC, vascular endothelial growth factor receptor 2, and MAP kinase p38 alpha. The reason for this was unclear. When a target protein has multiple ligand binding sites, such as orthosteric and allosteric sites, the linear regression model is not suitable. In this case, nonlinear regression, such as logistic regression and neural networks, may solve the problem. But the present model is based on eqs. 2–4. When the target protein structure gave the docking score without computational error, then ***Δ*** ***G***_***i***_^***a***^ *=**s***_***i***_^***a***^. The nonlinear regression must satisfy this simple condition. This problem is somewhat troublesome. We will examine it in the future.

The ChEMBL database did not provide details on the experimental conditions (density of native ligand, temperature, etc.). The ***Δ*** ***G*** values should be precise by using the actual experimental information. But this method requires natural language analysis, which is an expensive approach. One possible improvement of the present method might be batch effect correction.^52^ That is, we could determine some optimal artificial values as the experimental parameters, such as the densities of ligands and substrates, in order to minimize the prediction error. In this approach, first the present method generates a prediction model based on the experimental data with the standard experimental values, then the batch‐effect correction method would optimize the experimental parameters of each batch to minimize the computational error of the set of data points of the batch.

The outlier elimination improved accuracy more than the M‐estimation did. This gap might be attributable to the fact that the standard deviations of ***Δ*** ***G*** values differ from each other among the different targets and different compound sets, while the ***W*** value is consistent throughout all the data. This difference could be the reason why the outlier elimination improved the results better than the M estimation.

### 3.3 Application to MMPs

**Figure 5:**
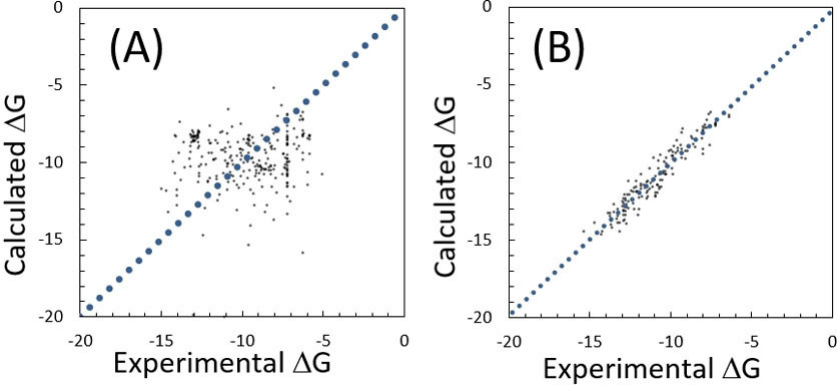
Correlation between experimental and prediction data for MMP2 (unit in kcal/mol). (A) obtained by the naïve protein−ligand docking calculation by Sievgene, and (B) obtained by the docking‐score QSAR model with λ=0.0002, W=20 kcal/mol. The dots represent the predicted data points by the LOO cross‐validation test.

We studied MMP2, MMP3, MMP7, MMP9, and MMP13 by the docking‐score QSAR method and the naïve protein−compound docking simulations. We applied the naïve protein−ligand docking calculation by Sievgene.^40^ Figure 5A shows the correlation between the experimental and the calculated protein−compound binding free energies of MMP2 by Sievgene. The averaged values of the ***Q*** ^***2***^ and RMSE were 0.037 and 1.692 kcal/mol, respectively. Table 5 summarizes the total number of ligands as well as the ***Q*** ^***2***^ and RMSE values of MMPs by naïve docking. The accuracy was poor and the results did not show any experimental trends, since the Sievgene docking program does not have a metal‐contact term to support the metal ions.

Next, we applied the docking‐score QSAR method to the same data sets. We applied the LOO cross‐validation test to the MMPs. Figure 5B shows the correlation between the experimental and calculated protein−compound binding free energies of MMP2 by eq. 8. The ***Q*** ^***2***^ and RMSE values by the LOO cross‐validation test were 0.88 and 1.08 kcal/mol, respectively. The RMSE values by the docking‐score QSAR method were much better than those by the naive docking, while both methods used the same docking program (Table 5).

We checked the individual correlation results. The results obtained by the docking‐score QSAR method were much better than those by the naïve docking in many cases. The overall trends of the results were the same as those in the kinase cases (Figure S3). Namely, the average RMSE value by the naïve docking studies was 1.7 kcal/mol. These values were close to the deviation of the experimental ***Δ*** ***G*** values. On the other hand, the docking‐score QSAR showed an average RMSE of 1.1 kcal/mol and a better ***Q*** ^***2***^.

The docking‐score QSAR method used only the Sievgene docking scores and was exactly the same program used in the above naïve docking study without any parameter tuning for the quantum mechanical interaction between the metal and the ligand. The docking‐score QSAR method should take into account the quantum mechanical interaction implicitly to improve the prediction results. The RMSE values were similar to those given in Section 3.2.

The docking‐score QSAR method was based on the docking program without consideration of the quantum effect between the metal atom and the ligands, but it improved the RMSE by 0.6 kcal/mol, the same as in the kinase cases. Thus, the present method predicted ***Δ*** ***G*** values with an RMSE of 1 kcal/mol even for the difficult targets like the MMPs, and it could be applied to general target proteins.

The force‐field parameters for the metal−ligand interaction are generally poor, because such interactions should include quantum effects, which depend on the environment. Consequently, the naïve docking simulations could not provide good scores for the MMP systems. The current docking‐score QSAR model is based on a docking score that does not include a quantum effect, but this QSAR model is a completely different approach from that of the naive docking study. The machine learning procedures provided smaller RMSE value than the naive docking study.

## 4 Conclusions

We developed a docking‐score QSAR model based on combinations of multiple docking scores from protein−drug docking simulations, and applied this model to 107 kinase proteins from the ChEMBL public database. The prediction model employed a descriptor‐based weighted PCR with a regularization term and robust estimation (M estimation) methods in order to realize more realistic prediction than that by the ordinal multilinear regression model. The compound descriptor was a set of docking scores against many nontarget proteins. The LOO and 4‐fold cross‐validation tests showed that the addition of the regularization term improved the RMSE from 1.5 kcal/mol to 1.1 kcal/mol. In addition, the data preparation with outlier elimination worked to improve the results. We also applied the present method to MMPs that were difficult targets because of the quantum mechanical interaction. The present method improved the RMSE values for the MMPs without any manual parameter tuning, the same as in the kinase cases. The LOO cross‐validation tests showed that the docking‐score QSAR method improved the RMSE from 1.7 kcal/mol by the naïve docking calculation to 1.1 kcal/mol. These results suggested that this method is applicable to general target proteins. The analysis performed was based on the cross‐validation tests only, and there was no prospective experimental validation in this study. Further analysis of the validation tests designed for extrapolation should be performed.

## Supporting Information

The appendices, Figures S1–S3, and Tables S1–S5 were supplied as described in the Supporting Information.

## Abbreviations

QSAR: quantitative structure−activity relationship
LOO: leave‐one‐out
PCA: principal component analysis
PCR: principal component regression
RMSE: root‐mean‐square deviation
Q^2^: coefficient of determination of prediction

## Conflict of Interest

None declared.

## Supporting information

As a service to our authors and readers, this journal provides supporting information supplied by the authors. Such materials are peer reviewed and may be re‐organized for online delivery, but are not copy‐edited or typeset. Technical support issues arising from supporting information (other than missing files) should be addressed to the authors.

Supplementary
