# Supplementary material for: Prediction of Protein−compound Binding Energies from Known Activity Data: Docking‐score‐based Method and its Applications
Source: Mol Inform. 2018 Feb 14;37(6-7):1700120. doi: 10.1002/minf.201700120 (PMC6055825; doi:10.1002/minf.201700120)
Supplement: Supplementary file 1 — Supplementary [file MINF-37-na-s001.pdf]

Supporting Information

Prediction of protein−compound binding energies from known activity data: docking-score-based method and its applications

Yoshifumi Fukunishi,[a] Yasunobu Yamashita,[b] Tadaaki Mashimo,[b,c] and Haruki Nakamura[d]*

[a] Molecular Profiling Research Center for Drug Discovery (molprof), National Institute of Advanced Industrial Science and Technology (AIST), 2-3-26, Aomi, Koto-ku, Tokyo 135-0064, Japan

[b] Technology Research Association for Next-Generation Natural Products Chemistry, 2-3-26, Aomi, Koto-ku, Tokyo 135-0064, Japan

[c] IMSBIO Co., Ltd., Owl Tower. 4-21-1, Higashi-Ikebukuro, Toshima-ku, Tokyo 170-0013, Japan.

[d] Institute for Protein Research, Osaka University, 3-2 Yamadaoka, Suita, Osaka 565-0871, Japan
**e-mail: y-fukunishi@aist.go.jp, phone/fax: +81-3-3599-8290/+81-3-3599-8099*

**e-mail: y-fukunishi@aist.go.jp; phone/fax: +81-3-3599-8290/+81-3-3599-8099*

APPENDIX A

The selected 600 proteins were as follows: 10gs, 13gs, 17gs, 19gs, 1a0l, 1a27, 1a3k, 1a5h, 1aax, 1ad5, 1ae8, 1afe, 1agw, 1aht, 1aq1, 1aqw, 1aqx, 1atk, 1au0, 1au2, 1au3, 1au4, 1avn, 1awf, 1awh, 1axs, 1ayu, 1ayv, 1ayw, 1b09, 1b2y, 1b38, 1b39, 1b3d, 1b55, 1bhx, 1bic, 1bik, 1bio, 1biw, 1bj4, 1bkf, 1bl4, 1bl6, 1bl7, 1bmk, 1bmq, 1bpy, 1bui, 1bwn, 1byg, 1bzc, 1bzj, 1bzm, 1bzs, 1bzy, 1c1u, 1c1v, 1c1y, 1c4u, 1c4y, 1c5c, 1c5o, 1c5w, 1c5x, 1c5y, 1c8t, 1c9h, 1c9y, 1ca8, 1cam, 1can, 1caq, 1cay, 1caz, 1cbq, 1cbs, 1cc0, 1cd9, 1cea, 1ceb, 1ciz, 1ckp, 1cqp, 1ctr, 1d4p, 1d4x, 1d5j, 1d5m, 1d6v, 1d7h, 1d7i, 1d7j, 1d7x, 1d8f, 1db1, 1db4, 1db5, 1dcy, 1dhf, 1dhs, 1di8, 1di9, 1doa, 1doj, 1drf, 1dtw, 1dvs, 1dvt, 1dvu, 1dvx, 1dvy, 1dvz, 1dwb, 1dwc, 1dwd, 1e1v, 1e1x, 1e1z, 1e2d, 1e2q, 1e2s, 1e3c, 1e3k, 1e4h, 1e51, 1e5a, 1e7a, 1e7b, 1e7c, 1e7e, 1e7f, 1e7g, 1e7h, 1e7i, 1e87, 1e96, 1e9h, 1eax, 1ejn, 1ek5, 1ekf, 1ekp, 1ekv, 1emu, 1eou, 1ep9, 1equ, 1ere, 1err, 1exa, 1exx, 1f0r, 1f2w, 1f45, 1f5f, 1f5n, 1f9p, 1fak, 1fao, 1fby, 1fcx, 1fcy, 1fcz, 1fd0, 1fds, 1fdt, 1fdw, 1fgi, 1fhi, 1fin, 1fjs, 1fkb, 1fkd, 1fkf, 1fkg, 1fkh, 1fki, 1fkj, 1fls, 1fpc, 1fq1, 1fuj, 1fvo, 1fzc, 1fze, 1fzf, 1fzg, 1fzv, 1g05, 1g2l, 1g2m, 1g30, 1g32, 1g3m, 1g49, 1g4k, 1g5s, 1g7f, 1g7g, 1g86, 1g9n, 1gbn, 1gc1, 1gcq, 1gfw, 1gkc, 1gkd, 1gmn, 1gmo, 1gni,

1gos, 1gra, 1gs4, 1gua, 1guv, 1gv7, 1gwq, 1gwr, 1gyk, 1gzp, 1gzq, 1gzr, 1gzy, 1gzz, 1h07, 1h08, 1h0c, 1h0v, 1h0w, 1h1b, 1h1h, 1h1v, 1h1w, 1h2m, 1h2n, 1h2t, 1h2u, 1h52, 1h53, 1h8i, 1h9u, 1he2, 1he7, 1he8, 1hh8, 1hi3, 1hi4, 1hi5, 1i0z, 1i4d, 1i4l, 1i4t, 1i5r, 1i76, 1i7g, 1i7i, 1ibr, 1icf, 1iei, 1ii6, 1iiq, 1iis, 1iix, 1ikt, 1ikx, 1il0, 1iqe, 1iqf, 1iqg, 1iqh, 1iqj, 1iqk, 1iqm, 1iqn, 1ira, 1itq, 1itu, 1ivy, 1j1b, 1j1c, 1j78, 1j8h, 1j8u, 1j96, 1j99, 1jag, 1jbq, 1jbu, 1jdn, 1jdp, 1jdx, 1jgd, 1jiz, 1jj9, 1jk3, 1jk7, 1jk8, 1jkk, 1jqd, 1jqe, 1jsv, 1ju6, 1juj, 1jvp, 1jvq, 1jwh, 1jwt, 1k21, 1k22, 1k27, 1k3l, 1k5d, 1k8r, 1kak, 1kav, 1kbc, 1kcg, 1kdk, 1kfa, 1khb, 1khf, 1kjl, 1kjr, 1kkq, 1klt, 1kmv, 1knu, 1kq0, 1kts, 1ktt, 1kv1, 1kv2, 1kw0, 1kye, 1l2j, 1l8j, 1l8l, 1li4, 1lpk, 1ly2, 1m17, 1m27, 1m2z, 1m3h, 1m49, 1m4a, 1m4b, 1m4u, 1m51, 1m6w, 1m76, 1m7q, 1m9z, 1mc5, 1men, 1mkd, 1mmb, 1mmk, 1mmt, 1moo, 1mq5, 1mq6, 1mrq, 1msv, 1mu2, 1mu6, 1mu7, 1mu8, 1mu9, 1mue, 1mvs, 1mvt, 1mx1, 1mzf, 1n6c, 1n6h, 1n6k, 1n6l, 1n6n, 1n6o, 1n6p, 1n6r, 1n7i, 1n7j, 1n83, 1n86, 1n8z, 1nav, 1nax, 1nb3, 1nb5, 1nbp, 1nde, 1ne7, 1nf3, 1nf7, 1nhx, 1nhz, 1njs, 1nju, 1nkm, 1nl6, 1nl9, 1nlj, 1nlv, 1nm1, 1nm6, 1nmd, 1nmq, 1nmx, 1nmy, 1nmz, 1nn0, 1nn1, 1nn3, 1nn5, 1nn6, 1nny, 1no6, 1no9, 1now, 1np0, 1nrg, 1nrl, 1nsi, 1nt1, 1nun, 1nup, 1nvq, 1nvr, 1nvs, 1nwl, 1nwr, 1nws, 1nwt, 1nwu, 1ny3, 1nyx, 1nz7, 1nzq, 1o0d, 1o0v, 1o41, 1o42, 1o43, 1o44, 1o45, 1o46, 1o47, 1o48, 1o49, 1o4a, 1o4b, 1o4d, 1o4e, 1o4f, 1o4g, 1o4h, 1o4i, 1o4j, 1o4k, 1o4l, 1o4m, 1o4n, 1o4o, 1o4p, 1o4q, 1o4r, 1o5d, 1o5e, 1o5f, 1o6e, 1o6k, 1o6l, 1o6u, 1o86, 1o9s, 1oat, 1oec, 1of7, 1ogt, 1ogu, 1ohj, 1ohk, 1oi9, 1oiq, 1oir, 1oit, 1oiu, 1oiv, 1oiw, 1oix, 1ojb, 1ojc, 1ojd, 1okl, 1okm, 1okn, 1oky, 1okz, 1ol0, 1olm, 1ols, 1olu, 1olx, 1ony, 1onz, 1op3, 1oq5, 1osf, 1oth, 1ouk, 1ouy, 1ov4, 1ove, 1ovz, 1ow0, 1ow3, 1oyn, 1oyt, and 1oz1. For 10gs, 13gs, 17gs, 19gs, 1aax, 1ad5, 1ad8, 1aqw, 1aqx, 1avn, 1bb5, 1bic, 1bio, 1bpy, 1bzs, 1c5x, 1e2s, 1e7a, 1e7b, 1e7c, 1exa, 1exx, 1f5k, 1fak, 1fcx, 1fcy, 1fcz, 1fd0, 1fdt, 1fjs, 1fzc, 1g3m, 1g4k, 1g86, 1gc1, 1gni, 1gra, 1gzp, 1gzq, 1h07, 1h08x3, 1h0cx3, 1i5r, 1iei, 1ii6, 1itu, 1j78, 1jdp, 1li4, 1mc5, 1mrq, 1nrg, 1op3, and 1op3. In the case of certain proteins that bind two ligands, two pockets were prepared.

APPENDIX B

The ChEMBL database records the affinity data (IC_{50}, %-inhibition, %-residual activity, etc.) without experimental detail parameters, which are the densities of inhibitors and ligands. We must check the references to determine these parameters, or we must use standard values instead of the actual experimental values. In the present study, we used the standard values for the densities of the inhibitors and ligands. The protein−compound binding energy ΔG is calculated from the K_d value as follows:

$$\Delta G = k_B \cdot T \cdot \ln(K_d), \quad \text{eq. S1}$$

where k_B and T are the Boltzmann constant and temperature, respectively. In the present study, we assumed that $K_d = K_i$, since the binding affinities of the natural ligands have been reported to be much weaker than those of the reported artificial ligands in many proteins. In addition, the %inhibition value (r) gives the K_i values as follows in the present study.

$$K_i = \frac{[I]}{\frac{[S]/(1-r)-[S]}{K_s} - 1} \quad \text{eq. S2}$$

Here K_s, [S], and [I] are the rate constant, the density of the inhibitor, and the density of the substrate, respectively. The IC_{50} value is converted to the K_i value by the Cheng−Prusoff equation as follows.[32, 49]

$$K_i = \frac{IC_{50}}{1 + \frac{[S]}{K_s}} \quad \text{eq. S3}$$

As shown above, the ΔG value depends on the densities of the inhibitor, substrate, and K_s. The ChEMBL database does not provide these parameters explicitly. Instead of the true parameters described in each original experimental article, we used the same set of standard values for these parameters that we used in our previous work. Namely, [I] = 50 μM, [S] = 20 μM, and K_s = 1 μM in both eqs. S2 and S3, and [S]/K_s =1 in eq. S3.

Assay data with inadequate energy units or unclear energy values were excluded. The assay data for large compounds (mass weight > 500 Da) and very small compounds (mass weight < 150 Da) were also excluded, since Sievgene is designed for the docking of small compounds with mass weights > 150 Da and mass weights < 500 Da. In addition, we avoided the chemical compound structures that were not consistent with the same compounds registered in the PubChem database.

Table S1 Correlation (***R^2/Q^2***) between the experimental data and the calculated data obtained by the LOO cross validation test of the docking-score QSAR model over all 107 proteins.

Preferred name	No of ligands	$R^{2\ a}$	RMSEC (kcal/mol)[b]	$Q^{2\ c}$	RMSEP (kcal/mol)[d]
Tyrosine-protein kinase ABL1	795	0.67	0.96	0.57	1.11
Tyrosine-protein kinase ABL2	126	0.97	0.32	0.92	0.49
Serine/threonine-protein kinase AKT	833	0.74	1.50	0.67	1.70
Serine/threonine-protein kinase AKT2	286	0.94	0.73	0.86	1.12
Serine/threonine-protein kinase AKT3	102	0.96	0.41	0.92	0.62
ALK tyrosine kinase receptor	66	0.90	0.69	0.64	1.46
Serine/threonine-protein kinase Aurora-A	531	0.76	0.85	0.62	1.09
Serine/threonine-protein kinase Aurora-B	321	0.88	0.69	0.71	1.08
Serine/threonine-protein kinase Aurora-C	98	0.96	0.42	0.86	0.76
Serine/threonine-protein kinase B-raf	229	0.92	0.60	0.79	0.99
Cell division cycle 7-related protein kinase	185	0.97	0.22	0.81	0.55
Cyclin-dependent kinase 1	1582	0.47	1.41	0.32	1.60
Cyclin-dependent kinase 2	2223	0.40	1.65	0.25	1.86
Cyclin-dependent kinase 4	1261	0.61	1.10	0.48	1.26
Cyclin-dependent kinase 5	599	0.69	1.14	0.56	1.37
Cyclin-dependent kinase 9	86	0.91	0.49	0.77	0.82
Inhibitor of nuclear factor kappa B kinase alpha subunit	255	0.83	0.55	0.60	0.86
Dual specificity protein kinase CLK4	72	0.96	0.29	0.86	0.54
Macrophage colony stimulating factor receptor	420	0.73	1.04	0.59	1.30
Tyrosine-protein kinase CSK	130	0.86	0.69	0.57	1.24
Serine/threonine-protein kinase Chk1	928	0.63	1.53	0.55	1.70
Serine/threonine-protein kinase Chk2	211	0.92	0.73	0.77	1.32
Dual-specificity tyrosine-phosphorylation regulated kinase 2	69	0.97	0.31	0.88	0.64
Dual-specificity tyrosine-phosphorylation regulated kinase 3	57	0.99	0.19	0.83	0.67
Epidermal growth factor receptor erbB1	3181	1.00	1.00	0.30	2.01
MAP kinase ERK2	296	0.91	0.76	0.78	1.17
MAP kinase ERK1	218	0.94	0.60	0.81	1.08
Mitogen-activated protein kinase 15	73	0.98	0.20	0.86	0.59
Focal adhesion kinase 1	157	0.98	0.22	0.87	0.51
Fibroblast growth factor receptor 1	548	0.76	0.81	0.63	1.03
Fibroblast growth factor receptor 2	116	0.98	0.27	0.89	0.65
Fibroblast growth factor receptor 3	103	0.97	0.35	0.93	0.50
Fibroblast growth factor receptor 4	76	1.00	0.12	0.93	0.58
Tyrosine-protein kinase receptor FLT3	423	0.86	0.69	0.74	0.94
Glycogen synthase kinase-3 alpha	103	0.94	0.52	0.88	0.74
Glycogen synthase kinase-3 alpha	465	0.84	0.91	0.72	1.22

Glycogen synthase kinase-3 beta	1522	0.50	1.58	0.38	1.76
Tyrosine-protein kinase HCK	208	0.94	0.42	0.82	0.73
Receptor protein-tyrosine kinase erbB-2	707	0.69	1.02	0.58	1.20
Homeodomain-interacting protein kinase 2	64	0.96	0.36	0.84	0.73
Homeodomain interacting protein kinase 3	61	0.99	0.15	0.83	0.83
Insulin-like growth factor I receptor	284	0.88	0.73	0.70	1.17
Inhibitor of nuclear factor kappa B kinase beta subunit	468	0.79	0.99	0.65	1.31
Insulin receptor	182	0.96	0.42	0.86	0.83
Interleukin-1 receptor-associated kinase 4	128	1.00	0.09	0.94	0.50
Tyrosine-protein kinase ITK/TSK	241	0.74	0.86	0.49	1.23
c-Jun N-terminal kinase 1	619	0.76	1.32	0.65	1.58
c-Jun N-terminal kinase 2	311	0.89	0.88	0.76	1.35
c-Jun N-terminal kinase 3	589	0.68	1.16	0.54	1.42
Stem cell growth factor receptor	615	0.78	0.84	0.68	1.03
Tyrosine-protein kinase LCK	1396	0.58	1.67	0.47	1.88
LIM domain kinase 2 isoform 2b	51	1.00	0.14	0.88	0.81
Tyrosine-protein kinase Lyn	185	0.79	1.35	0.52	2.11
MAP/microtubule affinity-regulating kinase 3	69	0.98	0.26	0.88	0.62
Dual specificity mitogen-activated protein kinase kinase 1	542	0.76	1.35	0.63	1.68
Maternal embryonic leucine zipper kinase	69	0.90	0.39	0.65	0.76
Hepatocyte growth factor receptor	325	0.88	0.80	0.72	1.23
MAP kinase-activated protein kinase 2	412	0.84	1.01	0.73	1.34
MAP kinase-interacting serine/threonine-protein kinase MNK1	89	0.96	0.41	0.82	0.87
Ribosomal protein S6 kinase alpha 5	89	0.98	0.32	0.83	0.86
Myosin light chain kinase, smooth muscle	123	0.94	0.52	0.87	0.80
NIMA (never in mitosis gene a)-related kinase 2	71	0.97	0.25	0.87	0.53
putative serine-threonine protein kinase	63	0.96	0.26	0.82	0.55
NIMA (never in mitosis gene a)-related kinase 7	62	0.99	0.14	0.92	0.38
Neurotrophic tyrosine kinase, receptor, type 1 isoform 1	130	0.63	1.43	0.26	2.04
Nerve growth factor receptor Trk-A	79	0.95	0.42	0.76	0.94
p21-activated kinase 6	68	0.98	0.28	0.87	0.73
Serine/threonine-protein kinase PAK7	71	0.99	0.17	0.94	0.41
Platelet-derived growth factor receptor alpha	433	0.75	1.00	0.58	1.34
Platelet-derived growth factor receptor beta	775	0.59	1.22	0.47	1.39
Phosphorylase kinase gamma subunit 1	104	0.98	0.34	0.94	0.62
Phosphorylase kinase gamma subunit 2	103	0.99	0.31	0.93	0.72
Serine/threonine-protein kinase PIM1	462	0.72	1.29	0.58	1.61
Serine/threonine-protein kinase PIM2	233	0.93	0.61	0.77	1.18
Serine/threonine-protein kinase PIM3	120	0.89	0.69	0.68	1.22
cAMP-dependent protein kinase alpha-catalytic subunit	383	0.86	0.93	0.76	1.27

cAMP-dependent protein kinase beta-1 catalytic subunit	308	0.89	0.88	0.77	1.32
cAMP-dependent protein kinase, gamma catalytic subunit	303	0.90	0.84	0.79	1.27
Protein kinase C alpha	669	0.71	1.26	0.61	1.49
Protein kinase C beta	441	0.83	0.82	0.71	1.06
Protein kinase C delta	273	0.90	0.67	0.79	1.01
Protein kinase C gamma	255	0.92	0.61	0.80	0.98
Protein kinase C eta	220	0.90	0.71	0.76	1.16
Protein kinase C iota	195	0.94	0.51	0.84	0.86
Protein kinase C theta	444	0.80	0.94	0.67	1.23
Protein kinase C zeta	218	0.89	0.84	0.74	1.28
Protein kinase C mu	213	0.91	0.75	0.79	1.18
Protein kinase C nu	147	0.94	0.54	0.86	0.82
Protein kinase N2	84	0.99	0.21	0.91	0.59
Serine/threonine-protein kinase PLK1	271	0.92	0.74	0.78	1.26
Serine/threonine-protein kinase RAF	501	0.80	1.11	0.68	1.40
Tyrosine-protein kinase receptor RET	91	0.92	0.44	0.78	0.72
Serine/threonine-protein kinase Sgk1	106	0.96	0.40	0.84	0.78
Tyrosine-protein kinase SRC	1740	0.53	1.47	0.36	1.72
Serine/threonine-protein kinase SRPK1	75	0.94	0.39	0.78	0.78
Serine/threonine-protein kinase MST1	85	0.98	0.26	0.85	0.79
Tyrosine-protein kinase SYK	311	0.87	0.65	0.72	0.98
Tyrosine-protein kinase TIE-2	540	0.80	0.78	0.68	1.00
Vascular endothelial growth factor receptor 1	539	0.72	0.80	0.57	0.99
Vascular endothelial growth factor receptor 2	3200	1.00	1.00	0.19	1.64
Vascular endothelial growth factor receptor 3	122	0.92	0.50	0.83	0.75
Serine/threonine-protein kinase WEE1	483	0.94	0.29	0.90	0.40
Tyrosine-protein kinase ZAP-70	68	0.99	0.27	0.94	0.63
MAP kinase p38 alpha	2647	1.00	1.00	0.26	1.81
MAP kinase p38 beta	553	0.75	1.20	0.62	1.49
MAP kinase p38 delta	403	0.81	1.06	0.66	1.43
MAP kinase p38 gamma	428	0.81	1.11	0.69	1.44

[a]: Coefficient of determination of calculation

[b]: RMSE in calculation

[c]: Coefficient of determination of prediction

[d]: RMSE in prediction

Table S2 Correlation (***R^2/Q^2***) between the experimental data and the calculated data obtained by the 4-fold cross validation test of the docking-score QSAR model over all 107 proteins.

Preferred name	4-fold cross validation			
	$R^{2\,a}$	RMSEC (kcal/mol)[b]	$Q^{2\,c}$	RMSEP (kcal/mol)[d]
Tyrosine-protein kinase ABL1	0.68	0.92	0.54	1.11
Tyrosine-protein kinase ABL2	1.00	0.06	0.74	0.91
Serine/threonine-protein kinase AKT	0.76	1.50	0.66	1.76
Serine/threonine-protein kinase AKT2	0.95	0.66	0.84	1.19
Serine/threonine-protein kinase AKT3	1.00	0.05	0.87	0.78
ALK tyrosine kinase receptor	1.00	0.03	0.62	1.57
Serine/threonine-protein kinase Aurora-A	0.77	0.81	0.62	1.05
Serine/threonine-protein kinase Aurora-B	0.92	0.53	0.77	0.91
Serine/threonine-protein kinase Aurora-C	1.00	0.06	0.76	1.06
Serine/threonine-protein kinase B-raf	0.98	0.34	0.85	0.86
Cell division cycle 7-related protein kinase	0.99	0.13	0.76	0.46
Cyclin-dependent kinase 1	0.50	1.37	0.42	1.47
Cyclin-dependent kinase 2	1.00	1.00	0.26	1.86
Cyclin-dependent kinase 4	0.62	1.07	0.54	1.17
Cyclin-dependent kinase 5	0.64	1.21	0.47	1.46
Cyclin-dependent kinase 9	0.99	0.21	0.81	0.74
Inhibitor of nuclear factor kappa B kinase alpha subunit	0.93	0.34	0.71	0.68
Dual specificity protein kinase CLK4	1.00	0.01	0.74	0.73
Macrophage colony stimulating factor receptor	0.85	0.75	0.68	1.10
Tyrosine-protein kinase CSK	1.00	0.05	0.79	0.86
Serine/threonine-protein kinase Chk1	0.65	1.52	0.57	1.68
Serine/threonine-protein kinase Chk2	0.97	0.48	0.79	1.22
Dual-specificity tyrosine-phosphorylation regulated kinase 2	1.00	0.03	0.65	1.03
Dual-specificity tyrosine-phosphorylation regulated kinase 3	1.00	0.02	0.82	0.64
Epidermal growth factor receptor erbB1	1.00	1.00	0.27	1.97
MAP kinase ERK2	0.92	0.73	0.80	1.10
MAP kinase ERK1	0.98	0.38	0.86	0.91
Mitogen-activated protein kinase 15	1.00	0.04	0.50	1.23
Focal adhesion kinase 1	0.99	0.10	0.84	0.56
Fibroblast growth factor receptor 1	0.78	0.78	0.61	1.03
Fibroblast growth factor receptor 2	1.00	0.08	0.76	0.99
Fibroblast growth factor receptor 3	1.00	0.09	0.79	0.93
Fibroblast growth factor receptor 4	1.00	0.04	0.81	0.96
Tyrosine-protein kinase receptor FLT3	0.89	0.61	0.73	0.94
Glycogen synthase kinase-3 alpha	0.99	0.16	0.61	1.32
Glycogen synthase kinase-3 alpha	0.82	0.96	0.68	1.31
Glycogen synthase kinase-3 beta	0.53	1.55	0.44	1.69
Tyrosine-protein kinase HCK	0.96	0.33	0.78	0.76

Receptor protein-tyrosine kinase erbB-2	0.72	0.96	0.58	1.19
Homeodomain-interacting protein kinase 2	1.00	0.05	0.59	1.28
Homeodomain interacting protein kinase 3	1.00	0.04	0.68	1.02
Insulin-like growth factor I receptor	0.98	0.26	0.81	0.89
Inhibitor of nuclear factor kappa B kinase beta subunit	0.82	0.92	0.68	1.24
Insulin receptor	0.98	0.28	0.79	1.04
Interleukin-1 receptor-associated kinase 4	1.00	0.12	0.82	0.87
Tyrosine-protein kinase ITK/TSK	0.99	0.15	0.74	0.90
c-Jun N-terminal kinase 1	0.77	1.28	0.65	1.60
c-Jun N-terminal kinase 2	0.93	0.73	0.79	1.27
c-Jun N-terminal kinase 3	0.66	1.18	0.48	1.45
Stem cell growth factor receptor	0.79	0.83	0.63	1.11
Tyrosine-protein kinase LCK	0.64	1.55	0.58	1.67
LIM domain kinase 2 isoform 2b	1.00	0.01	0.80	1.04
Tyrosine-protein kinase Lyn	0.99	0.23	0.81	1.25
MAP/microtubule affinity-regulating kinase 3	1.00	0.05	0.65	1.09
Dual specificity mitogen-activated protein kinase kinase 1	0.76	1.38	0.61	1.76
Maternal embryonic leucine zipper kinase	1.00	0.04	0.74	0.62
Hepatocyte growth factor receptor	0.92	0.65	0.72	1.22
MAP kinase-activated protein kinase 2	0.86	0.97	0.70	1.40
MAP kinase-interacting serine/threonine-protein kinase MNK1	1.00	0.05	0.86	0.79
Ribosomal protein S6 kinase alpha 5	1.00	0.07	0.77	1.03
Myosin light chain kinase, smooth muscle	0.99	0.18	0.82	0.97
NIMA (never in mitosis gene a)-related kinase 2	1.00	0.03	0.66	0.76
putative serine-threonine protein kinase	1.00	0.03	0.66	0.75
NIMA (never in mitosis gene a)-related kinase 7	1.00	0.04	0.60	0.89
Neurotrophic tyrosine kinase, receptor, type 1 isoform 1	1.00	0.09	0.65	1.03
Nerve growth factor receptor Trk-A	1.00	0.04	0.73	0.92
p21-activated kinase 6	1.00	0.04	0.86	0.67
Serine/threonine-protein kinase PAK7	1.00	0.04	0.59	1.03
Platelet-derived growth factor receptor alpha	0.75	1.03	0.55	1.38
Platelet-derived growth factor receptor beta	0.59	1.25	0.44	1.46
Phosphorylase kinase gamma subunit 1	1.00	0.06	0.86	0.99
Phosphorylase kinase gamma subunit 2	1.00	0.06	0.87	0.97
Serine/threonine-protein kinase PIM1	0.81	1.06	0.62	1.49
Serine/threonine-protein kinase PIM2	0.98	0.37	0.85	0.96
Serine/threonine-protein kinase PIM3	1.00	0.05	0.83	0.93
cAMP-dependent protein kinase alpha-catalytic subunit	0.87	0.93	0.75	1.28
cAMP-dependent protein kinase beta-1 catalytic subunit	0.92	0.77	0.75	1.37
cAMP-dependent protein kinase, gamma catalytic subunit	0.92	0.80	0.77	1.32
Protein kinase C alpha	0.78	1.14	0.67	1.39
Protein kinase C beta	0.87	0.73	0.73	1.06
Protein kinase C delta	0.96	0.46	0.80	0.98
Protein kinase C gamma	0.97	0.40	0.81	0.94
Protein kinase C eta	0.98	0.36	0.82	0.98
Protein kinase C iota	1.00	0.14	0.87	0.83

Protein kinase C theta	0.85	0.83	0.70	1.18
Protein kinase C zeta	0.97	0.41	0.83	1.04
Protein kinase C mu	0.97	0.40	0.74	1.32
Protein kinase C nu	1.00	0.14	0.81	0.97
Protein kinase N2	1.00	0.05	0.78	0.92
Serine/threonine-protein kinase PLK1	0.92	0.74	0.72	1.36
Serine/threonine-protein kinase RAF	0.82	1.07	0.67	1.43
Tyrosine-protein kinase receptor RET	1.00	0.05	0.67	0.86
Serine/threonine-protein kinase Sgk1	1.00	0.07	0.66	1.04
Tyrosine-protein kinase SRC	0.57	1.38	0.50	1.48
Serine/threonine-protein kinase SRPK1	1.00	0.03	0.72	0.90
Serine/threonine-protein kinase MST1	1.00	0.04	0.82	0.78
Tyrosine-protein kinase SYK	0.92	0.53	0.72	1.02
Tyrosine-protein kinase TIE-2	0.83	0.66	0.69	0.90
Vascular endothelial growth factor receptor 1	0.76	0.72	0.61	0.90
Vascular endothelial growth factor receptor 2	1.00	1.00	0.25	1.53
Vascular endothelial growth factor receptor 3	1.00	0.05	0.82	0.81
Serine/threonine-protein kinase WEE1	0.92	0.34	0.82	0.53
Tyrosine-protein kinase ZAP-70	1.00	0.03	0.84	1.11
MAP kinase p38 alpha	1.00	1.00	0.24	1.89
MAP kinase p38 beta	0.75	1.20	0.57	1.58
MAP kinase p38 delta	0.84	0.95	0.64	1.45
MAP kinase p38 gamma	0.84	1.03	0.69	1.43

[a]: Coefficient of determination of calculation

[b]: RMSE in calculation

[c]: Coefficient of determination of prediction

[d]: RMSE in prediction

Table S3 Redundancy and deviation of multiple experimental data in KinaseSARfari data

Preferred name	Ki/Kd		IC_{50}		%-inhibition/%-activity		ΔG	
	No. of groups	σ (kcal /mol)	No. of groups	σ (kcal /mol)	No. of groups	σ (kcal/mol)	No. of groups	σ (kcal /mol)
Tyrosine-protein kinase ABL1	173	0.406	42	0.587	2	0.015	247	0.749
Tyrosine-protein kinase ABL2	22	0.262	0	ND	0	ND	29	0.514
Serine/threonine-protein kinase AKT	0	ND	135	0.694	12	0.132	210	0.986
Serine/threonine-protein kinase AKT2	0	ND	9	0.191	12	0.15	51	0.761
Serine/threonine-protein kinase AKT3	0	ND	7	0.145	0	ND	12	1.012
ALK tyrosine kinase receptor	0	ND	8	0.586	0	ND	20	0.968
Serine/threonine-protein kinase Aurora-A	11	0.268	33	0.573	4	0.003	74	0.841
Serine/threonine-protein kinase Aurora-B	10	0.448	19	0.524	12	0.081	71	0.699
Serine/threonine-protein kinase Aurora-C	16	0.068	0	ND	12	0.095	38	0.566
Serine/threonine-protein kinase B-raf	15	0.255	19	0.429	0	ND	56	0.687
Cell division cycle 7-related protein kinase	0	ND	11	0.151	0	ND	38	0.24
Cyclin-dependent kinase 1	4	0.084	244	0.272	36	0.164	315	0.394
Cyclin-dependent kinase 2	11	0.187	370	0.287	69	0.106	502	0.536
Cyclin-dependent kinase 4	2	0.098	193	0.28	38	0.134	266	0.503
Cyclin-dependent kinase 5	8	0.221	42	0.131	16	0.154	74	0.782
Cyclin-dependent kinase 9	0	ND	17	0.436	0	ND	22	0.756
Inhibitor of nuclear factor kappa B kinase alpha subunit	0	ND	27	0.134	0	ND	31	0.719
Dual-specificity protein kinase CLK4	12	0.18	10	0.12	0	ND	27	0.371
Macrophage colony stimulating factor receptor	0	ND	61	0.824	17	0.404	105	1.277
Tyrosine-protein kinase CSK	8	0.099	0	ND	29	0.161	46	0.951
Serine/threonine-protein kinase Chk1	0	ND	30	0.269	18	0.202	141	1.094
Serine/threonine-protein kinase Chk2	0	ND	14	0.55	10	0.096	29	1.154
Dual-specificity tyrosine-phosphorylation regulated kinase 2	0	ND	0	ND	10	0.108	11	0.748
Dual-specificity tyrosine-phosphorylation regulated kinase 3	0	ND	0	ND	10	0.151	12	0.39

Epidermal growth factor receptor erbB1	74	0.379	845	0.354	72	0.247	1377	0.708
MAP kinase ERK2	0	ND	9	0.707	16	0.049	30	0.783
MAP kinase ERK1	0	ND	9	0.246	14	0.123	30	0.894
Mitogen-activated protein kinase 15	0	ND	0	ND	10	0.136	13	0.463
Focal adhesion kinase 1	6	0.053	17	0.249	0	ND	24	0.33
Fibroblast growth factor receptor 1	10	0.199	90	0.243	2	0.036	125	0.434
Fibroblast growth factor receptor 2	8	0.122	4	0.323	0	ND	18	0.511
Fibroblast growth factor receptor 3	18	0.22	2	0.685	0	ND	22	0.651
Fibroblast growth factor receptor 4	0	ND	0	ND	0	ND	2	0.746
Tyrosine-protein kinase receptor FLT3	79	0.253	21	0.301	2	0.067	123	0.568
Glycogen synthase kinase-3 alpha	13	0.246	13	0.741	0	ND	32	0.819
Glycogen synthase kinase-3 alpha	0	ND	12	0.395	8	0.084	32	0.747
Glycogen synthase kinase-3 beta	5	0.111	166	0.25	26	0.081	297	0.696
Tyrosine-protein kinase HCK	9	0.249	0	ND	0	ND	14	0.831
Receptor protein-tyrosine kinase erbB-2	6	0.389	142	0.386	38	0.146	205	0.569
Homeodomain-interacting protein kinase 2	0	ND	0	ND	10	0.133	10	0.83
Homeodomain-interacting protein kinase 3	0	ND	0	ND	10	0.074	6	0.429
Insulin-like growth factor I receptor	0	ND	116	0.481	2	0.078	134	0.701
Inhibitor of nuclear factor kappa B kinase beta subunit	0	ND	56	0.353	10	0.015	70	0.623
Insulin receptor	8	0.146	7	0.145	6	0.034	30	0.538
Interleukin-1 receptor-associated kinase 4	0	ND	0	ND	0	ND	0	ND
Tyrosine-protein kinase ITK/TSK	0	ND	122	0.782	0	ND	134	1.051
c-Jun N-terminal kinase 1	6	0.102	32	0.15	14	0.045	72	0.767
c-Jun N-terminal kinase 2	2	0.069	27	0.382	12	0.07	56	0.776
c-Jun N-terminal kinase 3	11	0.135	25	0.131	16	0.097	67	0.851
Stem cell growth factor receptor	73	0.532	175	0.109	2	0.033	274	0.442
Tyrosine-protein kinase LCK	29	0.169	224	0.947	2	0.071	331	1.131
LIM domain kinase 2 isoform 2b	0	ND	0	ND	0	ND	2	0.692
Tyrosine-protein kinase Lyn	12	0.19	20	2.475	0	ND	50	1.965
MAP/microtubule affinity-regulating kinase 3	3	0.092	0	ND	8	0.099	13	0.477
Dual specificity mitogen-activated protein kinase kinase 1	0	ND	72	0.429	10	0.106	99	0.71

Maternal embryonic leucine zipper kinase	0	ND	0	ND	10	0.153	13	0.816
Hepatocyte growth factor receptor	0	ND	49	0.269	2	0.063	64	0.426
MAP kinase-activated protein kinase 2	4	0.262	29	0.446	16	0.104	45	0.715
MAP kinase-interacting serine/threonine-protein kinase MNK1	0	ND	0	ND	10	0.044	11	0.997
Ribosomal protein S6 kinase alpha 5	13	0.132	0	ND	10	0.052	24	0.503
Myosin light chain kinase, smooth muscle	7	0.235	7	0.463	10	0.082	35	0.797
NIMA (never in mitosis gene a)-related kinase 2	8	0.199	0	ND	10	0.017	16	0.41
Putative serine-threonine protein kinase	0	ND	0	ND	8	0.039	6	0.774
NIMA (never in mitosis gene a)-related kinase 7	0	ND	0	ND	10	0.019	8	0.343
Neurotrophic tyrosine kinase, receptor, type 1 isoform 1	9	0.149	2	0.1	0	ND	32	2.268
Nerve growth factor receptor Trk-A	2	0.053	0	ND	8	0.114	16	0.765
p21-activated kinase 6	0	ND	0	ND	8	0.053	10	0.567
Serine/threonine-protein kinase PAK7	2	0.2	0	ND	10	0.093	12	0.338
Platelet-derived growth factor receptor alpha	0	ND	31	0.253	0	ND	43	0.599
Platelet-derived growth factor receptor beta	14	0.17	120	0.443	2	0.024	171	0.608
Phosphorylase kinase gamma subunit 1	10	0.353	0	ND	12	0.05	31	0.523
Phosphorylase kinase gamma subunit 2	6	0.301	0	ND	12	0.05	27	0.518
Serine/threonine-protein kinase PIM1	11	0.251	26	0.81	74	0.183	161	0.978
Serine/threonine-protein kinase PIM2	6	0.253	15	0.691	36	0.086	63	0.534
Serine/threonine-protein kinase PIM3	0	ND	0	ND	38	0.203	57	0.936
cAMP-dependent protein kinase alpha-catalytic subunit	0	ND	23	0.407	14	0.158	54	0.902
cAMP-dependent protein kinase beta-1 catalytic subunit	0	ND	18	0.44	2	0.066	27	0.847
cAMP-dependent protein kinase, gamma catalytic subunit	0	ND	18	0.44	2	0.066	26	0.743
Protein kinase C alpha	42	0.352	56	0.488	22	0.128	175	0.781
Protein kinase C beta	27	0.471	47	0.637	2	0.055	135	0.701
Protein kinase C delta	19	0.512	44	0.548	2	0.055	80	0.75
Protein kinase C gamma	23	0.26	38	0.55	2	0.055	73	0.682
Protein kinase C eta	10	0.809	32	0.591	2	0.055	59	0.961

Protein kinase C iota	0	ND	20	0.765	24	0.154	70	0.634
Protein kinase C theta	2	0.023	35	0.589	2	0.055	90	0.662
Protein kinase C zeta	0	ND	20	0.708	12	0.023	50	1.205
Protein kinase C mu	2	0.023	24	0.613	12	0.214	61	1.018
Protein kinase C nu	2	0.023	19	0.714	2	0.055	35	0.983
Protein kinase N2	0	ND	3	0.336	8	0.156	10	0.581
Serine/threonine-protein kinase PLK1	0	ND	12	0.313	10	0.058	24	0.83
Serine/threonine-protein kinase RAF	3	0.748	59	0.365	4	0.023	76	0.788
Tyrosine-protein kinase receptor RET	27	0.142	0	ND	0	ND	34	0.496
Serine/threonine-protein kinase Sgk1	0	ND	6	0.719	12	0.037	19	0.868
Tyrosine-protein kinase SRC	78	0.095	146	0.472	4	0.033	336	1.038
Serine/threonine-protein kinase SRPK1	0	ND	0	ND	12	0.17	15	0.689
Serine/threonine-protein kinase MST1	0	ND	0	ND	10	0.199	19	0.511
Tyrosine-protein kinase SYK	4	0.271	20	0.371	0	ND	63	0.657
Tyrosine-protein kinase TIE-2	7	0.145	140	0.108	24	0.097	216	0.356
Vascular endothelial growth factor receptor 1	0	ND	53	0.297	2	0.004	73	0.72
Vascular endothelial growth factor receptor 2	28	0.685	986	0.22	42	0.058	1273	0.51
Vascular endothelial growth factor receptor 3	14	0.194	7	0.46	2	0.03	34	0.858
Serine/threonine-protein kinase WEE1	0	ND	400	0.012	0	ND	400	0.016
Tyrosine-protein kinase ZAP-70	13	0.104	0	ND	0	ND	28	0.322
MAP kinase p38 alpha	71	0.347	338	0.276	16	0.1	763	0.621
MAP kinase p38 beta	14	0.281	27	0.312	12	0.102	70	0.7
MAP kinase p38 delta	5	0.349	17	0.301	12	0.066	40	1.008
MAP kinase p38 gamma	13	0.413	18	0.302	14	0.05	48	0.996
Average	10.8	0.243	59.8	0.439	10.9	0.093	106.9	0.729

“ND” means that there is no redundant data.

Table S4. Average correlation values (***R*^{2}/*Q*^{2}**) between the experimental data and the calculated data and RMSE obtained by the docking-score QSAR model with various *N* (*W*=20 and λ=0.002) for all 107 proteins.

		Regression		4-fold cross validation	
Nσ	Total no. of compounds [a]	R^{2} [a]	RMSEC (kcal/mol)[b]	Q^{2} [c]	RMSEP (kcal/mol)[d]
0.2σ	35050	0.912	0.471	0.675	1.152
0.4σ	35736	0.911	0.475	0.675	1.155
0.5σ	36249	0.910	0.480	0.675	1.155
0.6σ	36804	0.909	0.484	0.676	1.155
0.8σ	38124	0.908	0.489	0.685	1.134
σ	44063	0.882	0.645	0.644	1.206
2σ	45549	0.874	0.697	0.631	1.231
3σ	45650	0.871	0.704	0.630	1.235

[a]: The standard deviation of the whole observed data (kcal/mol)

[a]: Coefficient of determination of calculation

[b]: RMSE in calculation

[c]: Coefficient of determination of prediction

[d]: RMSE in prediction

Table S5. ***Q*²** values for selected kinases. The docking-score QSAR method (4-fold cross validation tests) and the myPresto/sievgene docking program with using the default parameters give the ***Q*²** and RMSEP values.

Preferred name	Kinase ID	PDB ID	QSAR model		Docking score	
			$Q^{2\ a}$	RMSEP (kcal/mol) [b]	$Q^{2\ a}$	RMSEP (kcal/mol)[b]
Tyrosine-protein kinase ABL1	hABL1_1950	3QRI	0.54	1.11	0.03	1.42
Tyrosine-protein kinase ABL2	hABL2_1942	3HMI	0.74	0.91	0.15	1.26
Serine/threonine-protein kinase AKT	hAKT1_2014	3MV5	0.66	1.76	0.19	1.73
Serine/threonine-protein kinase AKT2	hAKT2_2120	-	0.84	1.19	-	-
Serine/threonine-protein kinase AKT3	hAKT3_2128	-	0.87	0.78	-	-
ALK tyrosine kinase receptor	hALK_949	5FTO	0.62	1.57	0.14	2.06
Serine/threonine-protein kinase Aurora-A	hAURa_2362	4BYI	0.62	1.05	0.02	1.23
Serine/threonine-protein kinase Aurora-B	hAURb_2346	-	0.77	0.91	-	-
Serine/threonine-protein kinase Aurora-C	hAURc_2382	-	0.76	1.06	-	-
Serine/threonine-protein kinase B-raf	hBRAF_1421	5FD2	0.85	0.86	0.06	1.26
Cell division cycle 7-related protein kinase	hCDC7_4	4F99	0.76	0.46	0.02	1.37
Cyclin-dependent kinase 1	hCDK1_416	-	0.42	1.47		
Cyclin-dependent kinase 2	hCDK2_583	1DM2	0.26	1.86	0.04	1.63
Cyclin-dependent kinase 4	hCDK4_340	-	0.54	1.17	-	-
Cyclin-dependent kinase 5	hCDK5_607	-	0.47	1.46	-	-
Cyclin-dependent kinase 9	hCDK9_246	-	0.81	0.74	-	-
Inhibitor of nuclear factor kappa B kinase alpha subunit	hCHUK_513	-	0.71	0.68	-	-
Dual specificity protein kinase CLK4	hCLK4_128	-	0.74	0.73	-	-
Macrophage colony stimulating factor receptor	hCSF1R_55	-	0.68	1.10	-	-
Tyrosine-protein kinase CSK	hCSK_2470	1BYG	0.79	0.86	0.24	1.76
Serine/threonine-protein kinase Chk1	hChk1_1758	2E9P	0.57	1.68	0.19	1.50
Serine/threonine-protein kinase Chk2	hChk2_1009	2W7X	0.79	1.22	0.15	1.99

Dual-specificity tyrosine-phosphorylation regulated kinase 2	hDYRK2_173	4AZF	0.65	1.03	0.03	2.01
Dual-specificity tyrosine-phosphorylation regulated kinase 3	hDYRK3_167	-	0.82	0.64	-	-

[a]: Coefficient of determination of prediction

[b]: RMSE in prediction

Figure S1 ***W*** dependence of $\boldsymbol{Q^2}$ and RMSEP. Blue and orange lines represent λ=0.002 and 0.005, respectively.

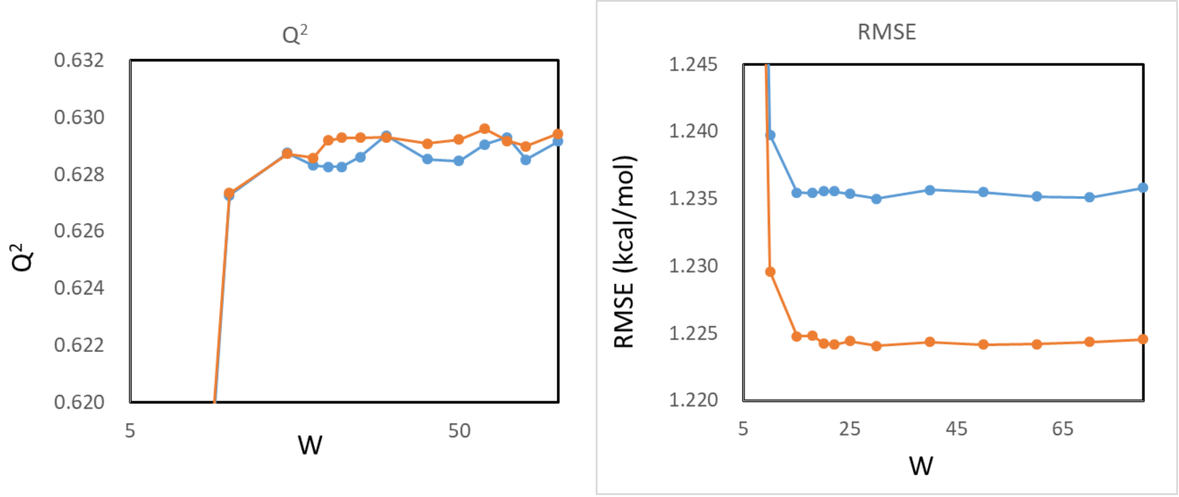

Figure S2 Correlation between the calculated and experimental ΔG values. The 4-fold cross validation results by the docking score QSAR method (λ=0.005, W=20, N=0.8) are in the left column. The naive docking results are in the right column (shown as „_by_docking“). The orage dotted lines are the y=x lines.

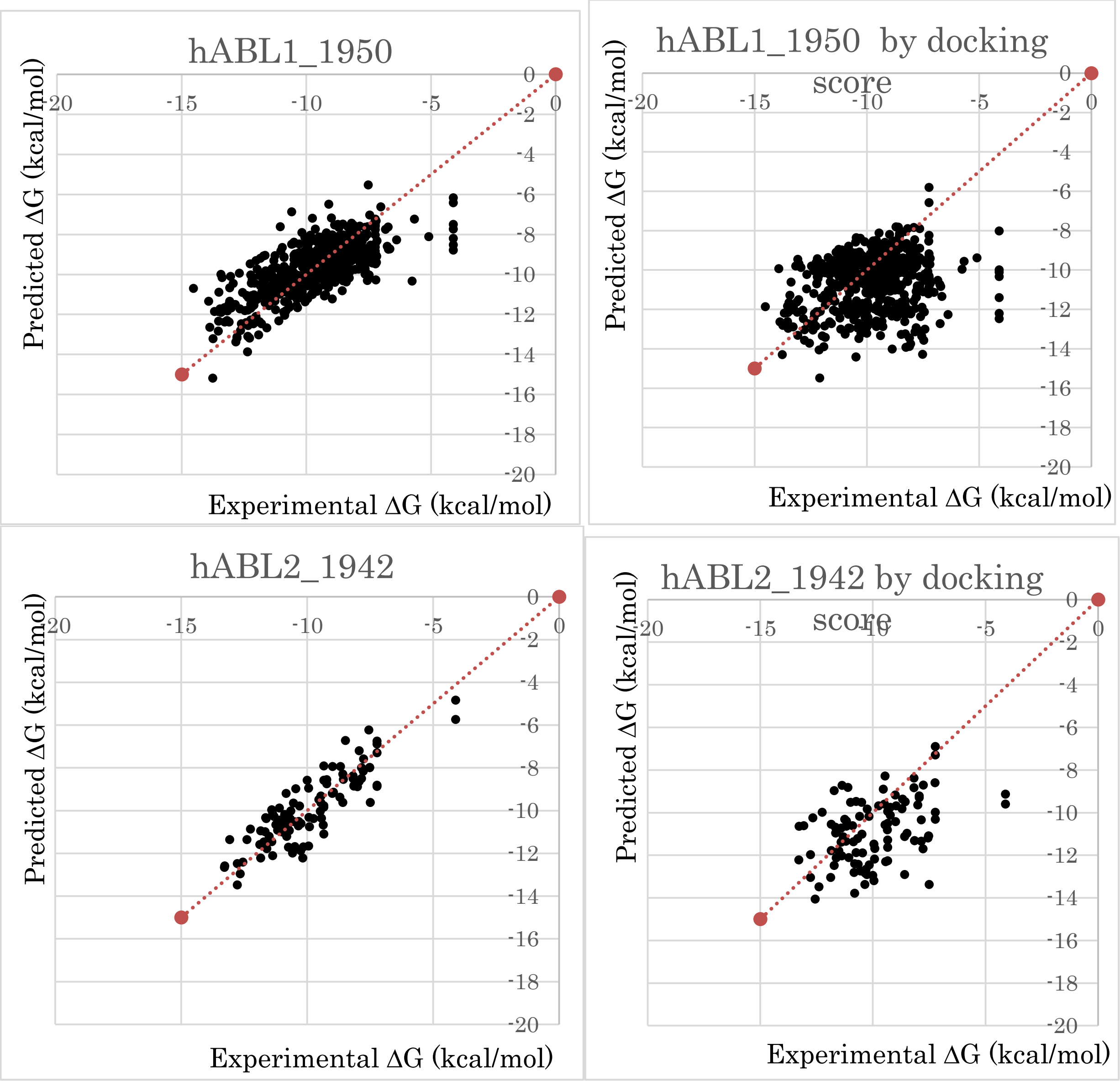

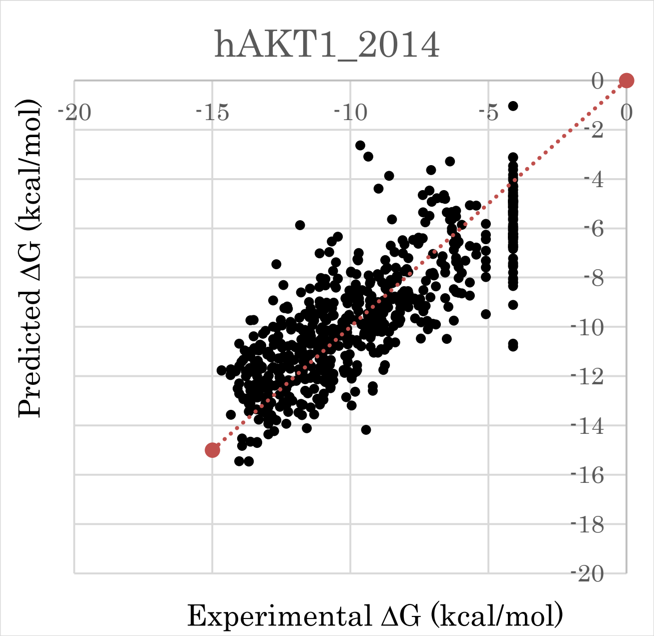
hAKT1_2014
Predicted ΔG (kcal/mol)
Experimental ΔG (kcal/mol)
-20
-15
-10
-5
0
-2
-4
-6
-8
-10
-12
-14
-16
-18
-20

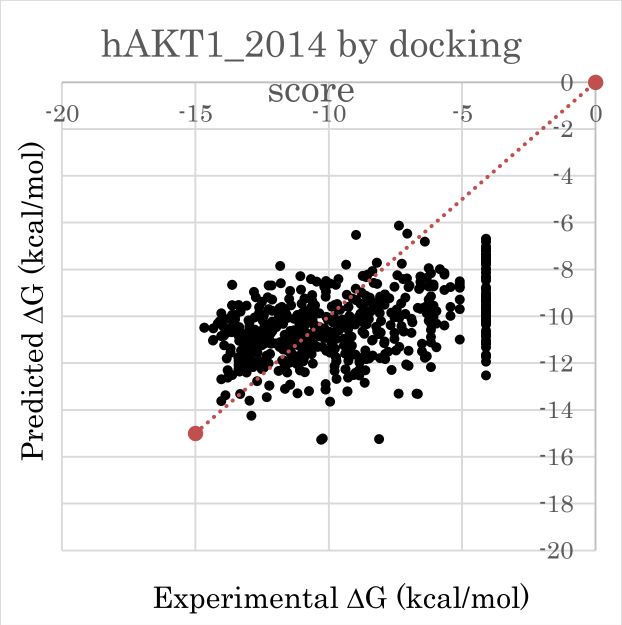
hAKT1_2014 by docking score
Predicted ΔG (kcal/mol)
Experimental ΔG (kcal/mol)
-20
-15
-10
-5
0
-2
-4
-6
-8
-10
-12
-14
-16
-18
-20

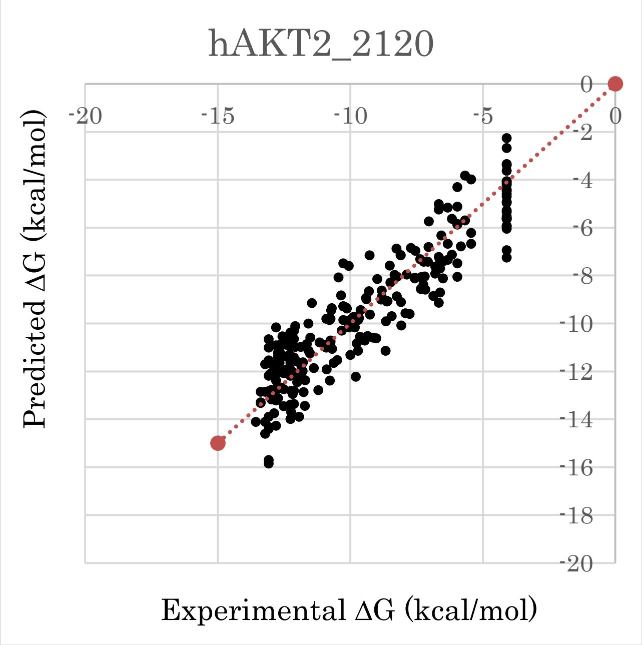
hAKT2_2120
Predicted ΔG (kcal/mol)
Experimental ΔG (kcal/mol)
-20
-15
-10
-5
0
-2
-4
-6
-8
-10
-12
-14
-16
-18
-20

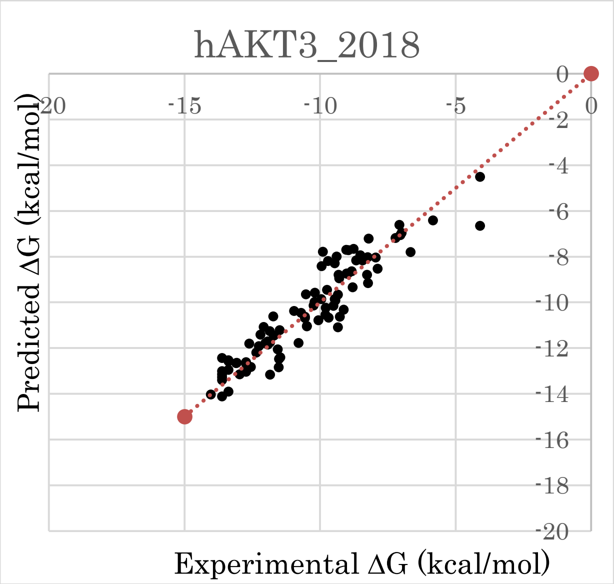
hAKT3_2018
Predicted ΔG (kcal/mol)
Experimental ΔG (kcal/mol)
-20
-15
-10
-5
0
-2
-4
-6
-8
-10
-12
-14
-16
-18
-20

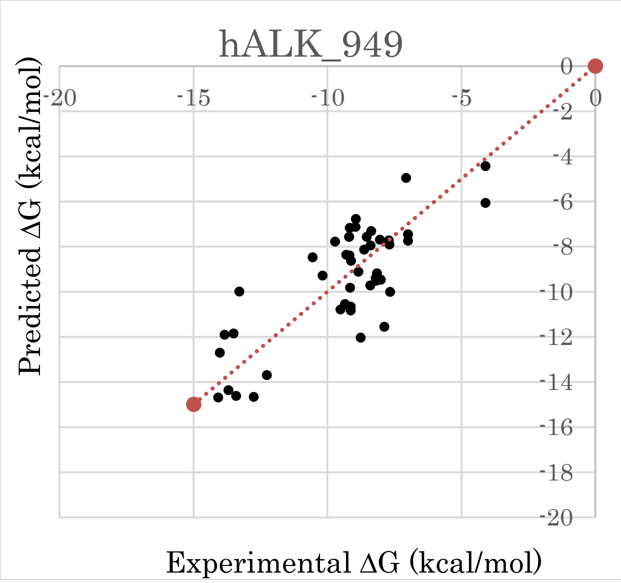
hALK_949
Predicted ΔG (kcal/mol)
Experimental ΔG (kcal/mol)
-20 -15 -10 -5 0
0 -2 -4 -6 -8 -10 -12 -14 -16 -18 -20

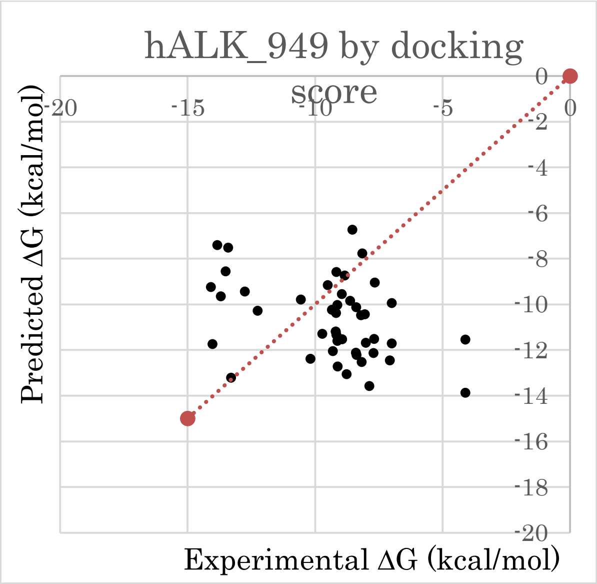
hALK_949 by docking score
Predicted ΔG (kcal/mol)
Experimental ΔG (kcal/mol)
-20 -15 -10 -5 0
0 -2 -4 -6 -8 -10 -12 -14 -16 -18 -20

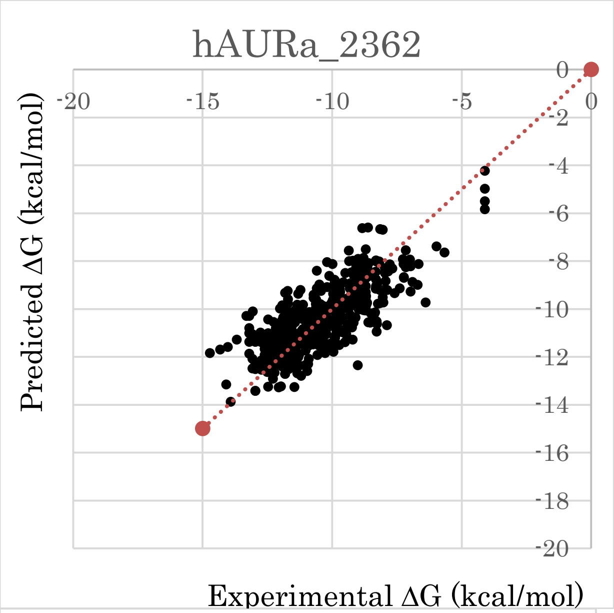
hAURa_2362
Predicted ΔG (kcal/mol)
Experimental ΔG (kcal/mol)
-20 -15 -10 -5 0
0 -2 -4 -6 -8 -10 -12 -14 -16 -18 -20

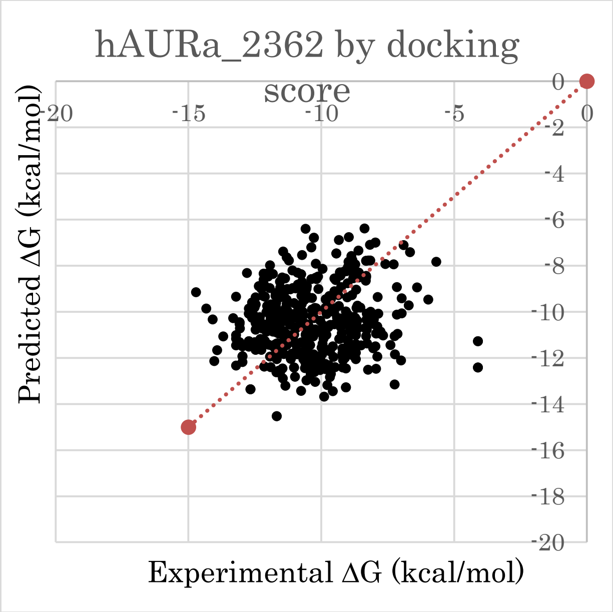
hAURa_2362 by docking score
Predicted ΔG (kcal/mol)
Experimental ΔG (kcal/mol)
-20 -15 -10 -5 0
0 -2 -4 -6 -8 -10 -12 -14 -16 -18 -20

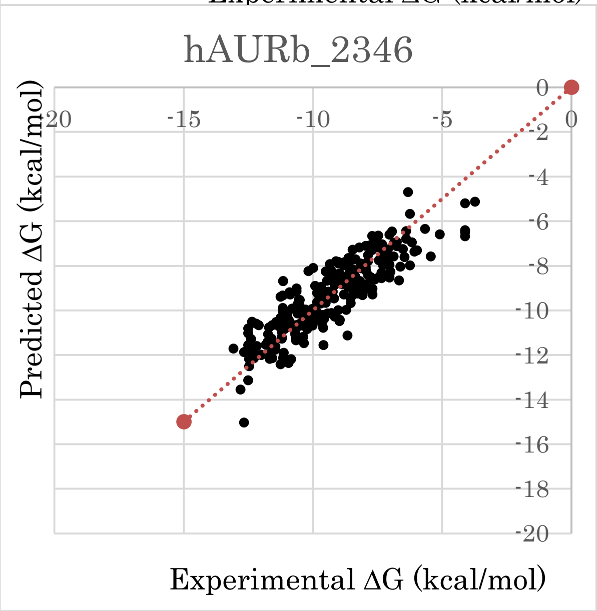
hAURb_2346
Predicted ΔG (kcal/mol)
Experimental ΔG (kcal/mol)
-20 -15 -10 -5 0
0 -2 -4 -6 -8 -10 -12 -14 -16 -18 -20

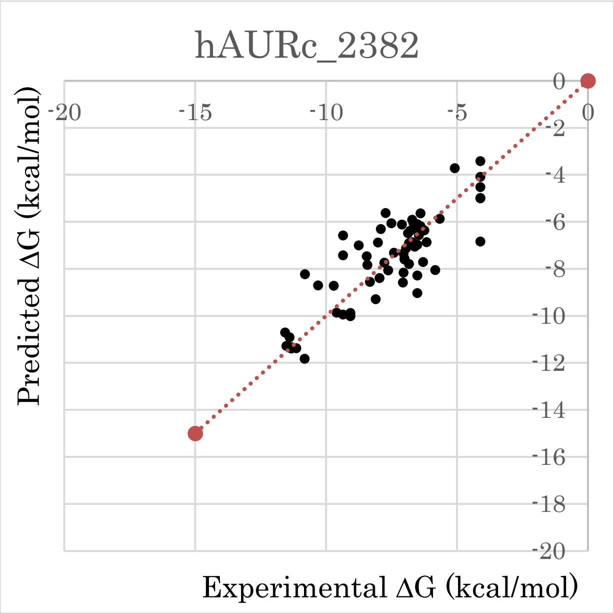
hAURc_2382
Predicted ΔG (kcal/mol)
Experimental ΔG (kcal/mol)
-20 -15 -10 -5 0
0 -2 -4 -6 -8 -10 -12 -14 -16 -18 -20

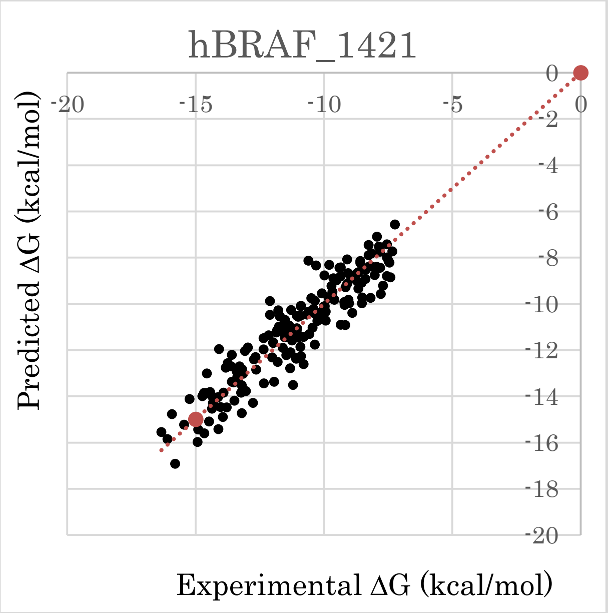
hBRAF_1421
Predicted ΔG (kcal/mol)
Experimental ΔG (kcal/mol)
-20 -15 -10 -5 0
0 -2 -4 -6 -8 -10 -12 -14 -16 -18 -20

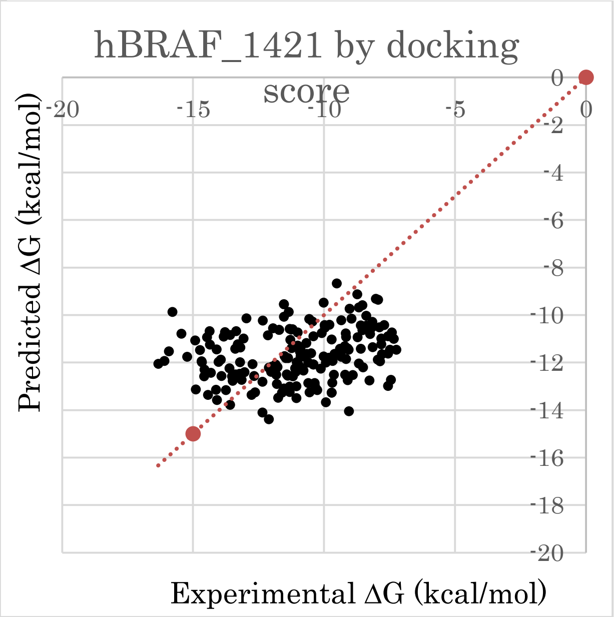
hBRAF_1421 by docking score
Predicted ΔG (kcal/mol)
Experimental ΔG (kcal/mol)
-20 -15 -10 -5 0
0 -2 -4 -6 -8 -10 -12 -14 -16 -18 -20

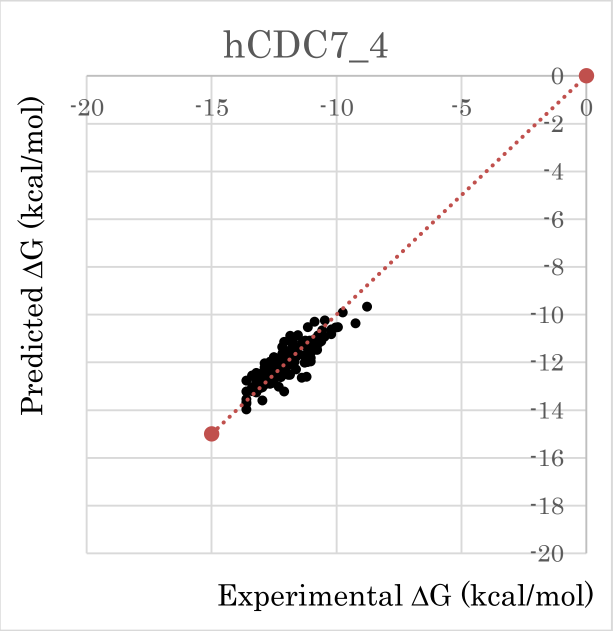
hCDC7_4
Predicted ΔG (kcal/mol)
Experimental ΔG (kcal/mol)
-20 -15 -10 -5 0
0 -2 -4 -6 -8 -10 -12 -14 -16 -18 -20

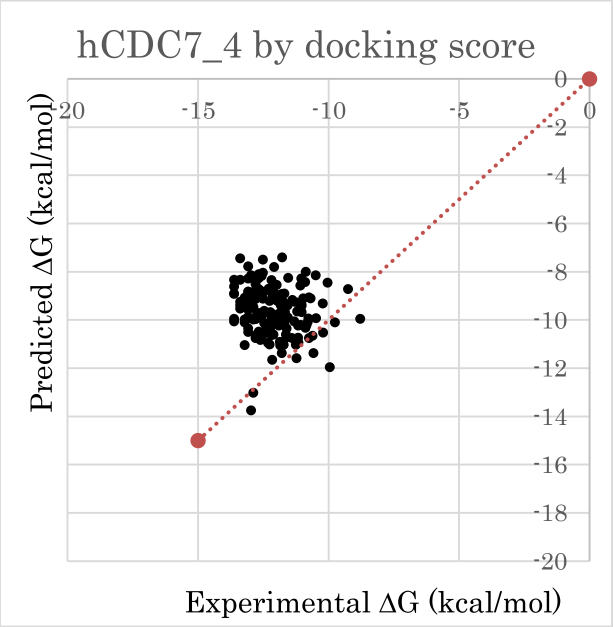
hCDC7_4 by docking score
Predicted ΔG (kcal/mol)
Experimental ΔG (kcal/mol)
-20 -15 -10 -5 0
0 -2 -4 -6 -8 -10 -12 -14 -16 -18 -20

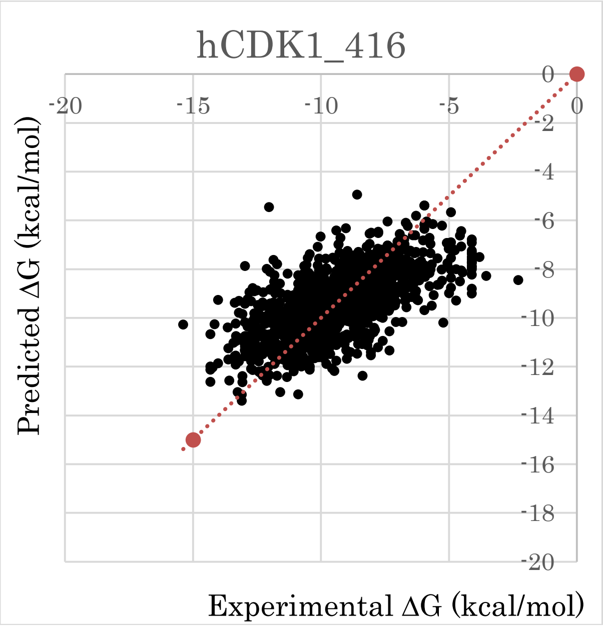
hCDK1_416
-20 -15 -10 -5 0
0 -2 -4 -6 -8 -10 -12 -14 -16 -18 -20
Predicted ΔG (kcal/mol)
Experimental ΔG (kcal/mol)

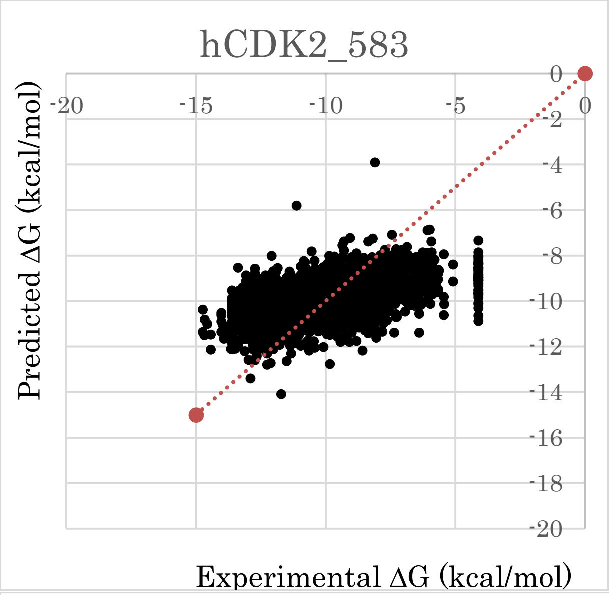
hCDK2_583
-20 -15 -10 -5 0
0 -2 -4 -6 -8 -10 -12 -14 -16 -18 -20
Predicted ΔG (kcal/mol)
Experimental ΔG (kcal/mol)

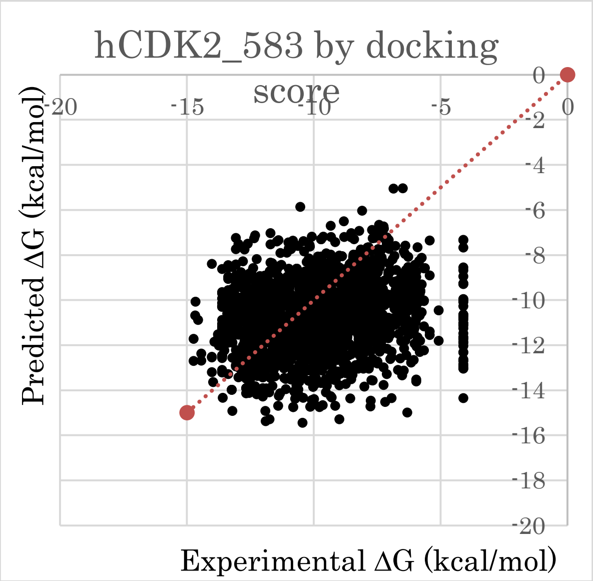
hCDK2_583 by docking score
-20 -15 -10 -5 0
0 -2 -4 -6 -8 -10 -12 -14 -16 -18 -20
Predicted ΔG (kcal/mol)
Experimental ΔG (kcal/mol)

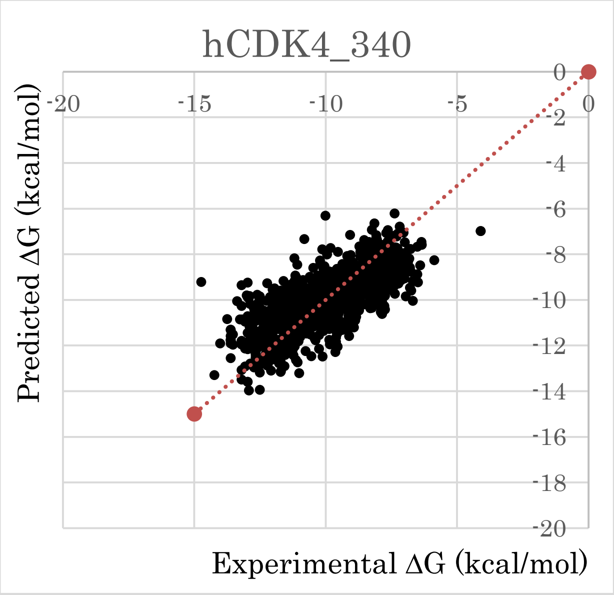
hCDK4_340
-20 -15 -10 -5 0
0 -2 -4 -6 -8 -10 -12 -14 -16 -18 -20
Predicted ΔG (kcal/mol)
Experimental ΔG (kcal/mol)

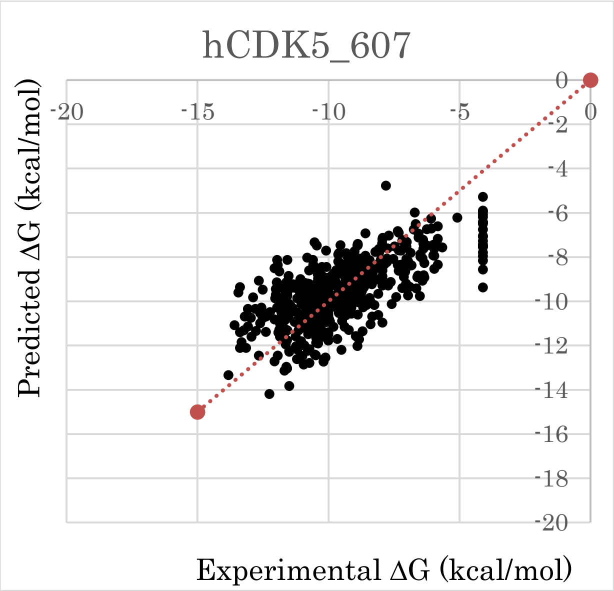
hCDK5_607
Predicted ΔG (kcal/mol)
Experimental ΔG (kcal/mol)
-20
-15
-10
-5
0
0
-2
-4
-6
-8
-10
-12
-14
-16
-18
-20

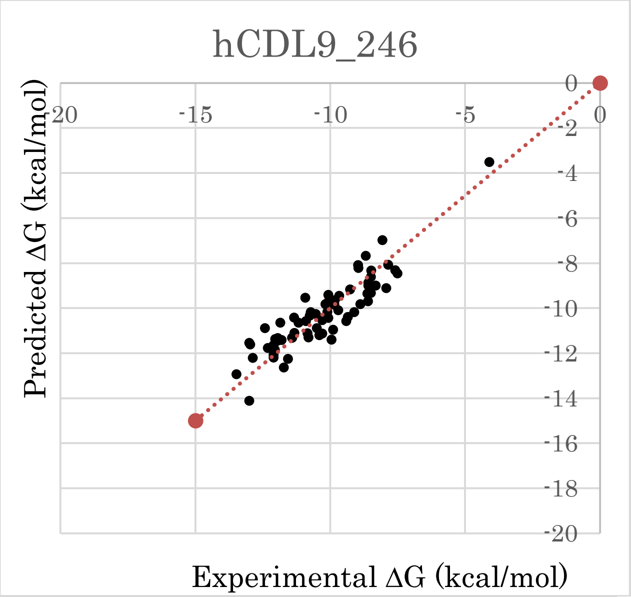
hCDL9_246
Predicted ΔG (kcal/mol)
Experimental ΔG (kcal/mol)
-20
-15
-10
-5
0
0
-2
-4
-6
-8
-10
-12
-14
-16
-18
-20

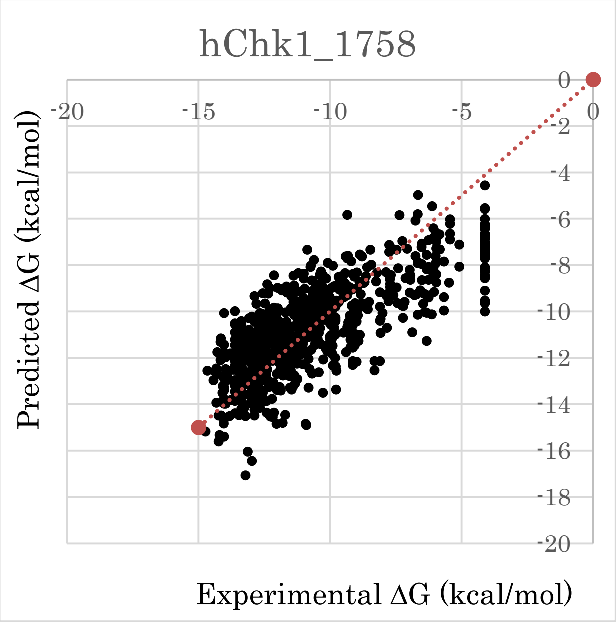
hChk1_1758
Predicted ΔG (kcal/mol)
Experimental ΔG (kcal/mol)
-20
-15
-10
-5
0
0
-2
-4
-6
-8
-10
-12
-14
-16
-18
-20

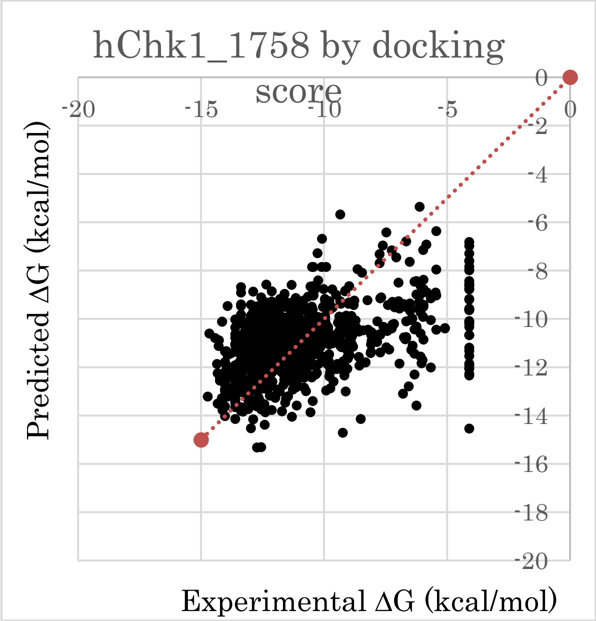
hChk1_1758 by docking score
Predicted ΔG (kcal/mol)
Experimental ΔG (kcal/mol)
-20
-15
-10
-5
0
0
-2
-4
-6
-8
-10
-12
-14
-16
-18
-20

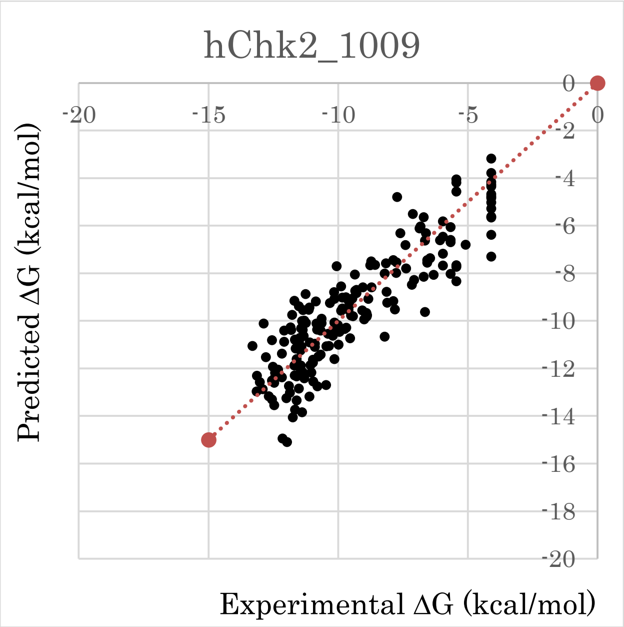
hChk2_1009
Predicted ΔG (kcal/mol)
Experimental ΔG (kcal/mol)
-20 -15 -10 -5 0
0 -2 -4 -6 -8 -10 -12 -14 -16 -18 -20

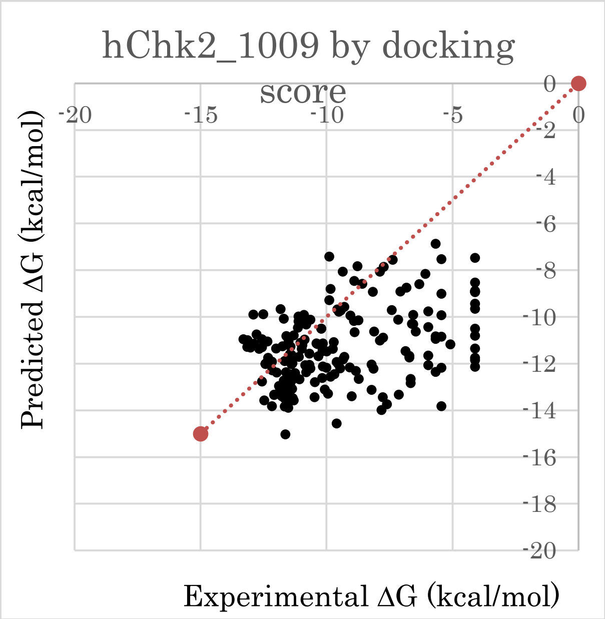
hChk2_1009 by docking score
Predicted ΔG (kcal/mol)
Experimental ΔG (kcal/mol)
-20 -15 -10 -5 0
0 -2 -4 -6 -8 -10 -12 -14 -16 -18 -20

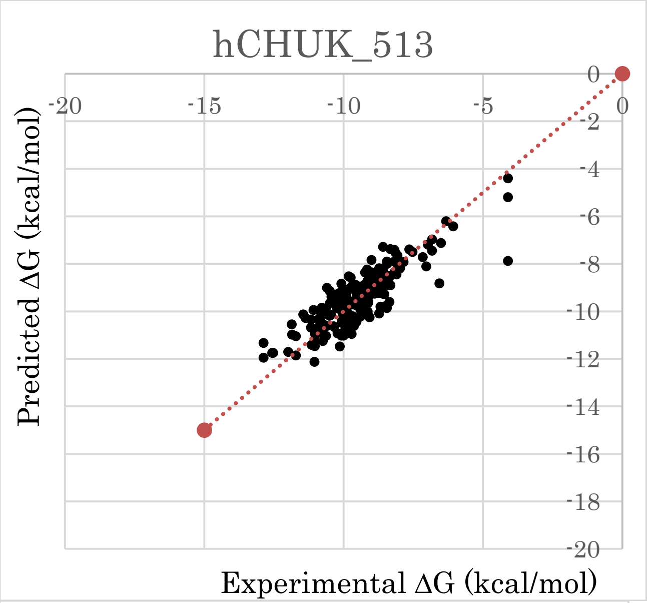
hCHUK_513
Predicted ΔG (kcal/mol)
Experimental ΔG (kcal/mol)
-20 -15 -10 -5 0
0 -2 -4 -6 -8 -10 -12 -14 -16 -18 -20

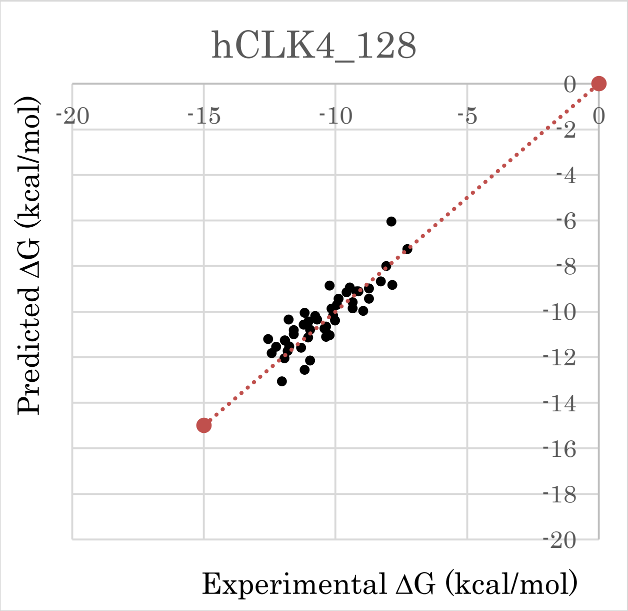
hCLK4_128
Predicted ΔG (kcal/mol)
Experimental ΔG (kcal/mol)
-20 -15 -10 -5 0
0 -2 -4 -6 -8 -10 -12 -14 -16 -18 -20

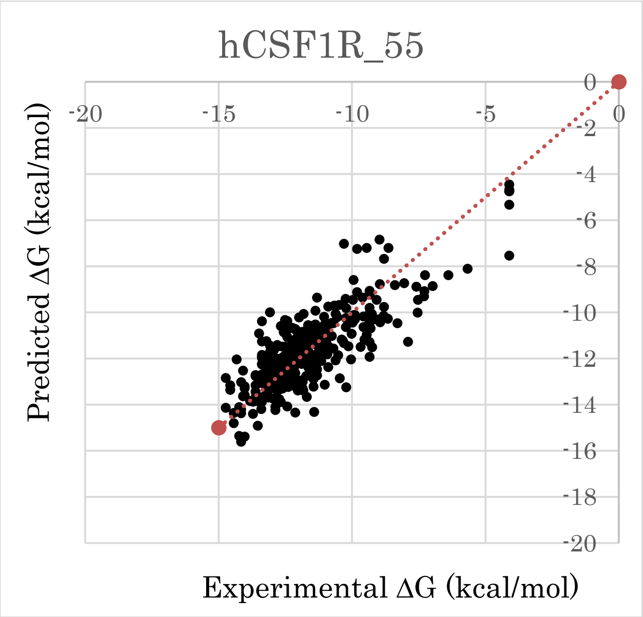
hCSF1R_55
Predicted ΔG (kcal/mol)
Experimental ΔG (kcal/mol)
-20
-15
-10
-5
0
-2
-4
-6
-8
-10
-12
-14
-16
-18
-20

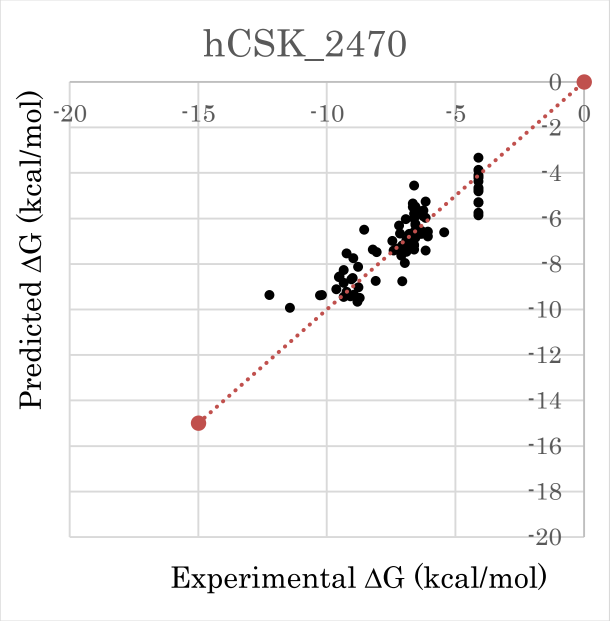
hCSK_2470
Predicted ΔG (kcal/mol)
Experimental ΔG (kcal/mol)
-20
-15
-10
-5
0
-2
-4
-6
-8
-10
-12
-14
-16
-18
-20

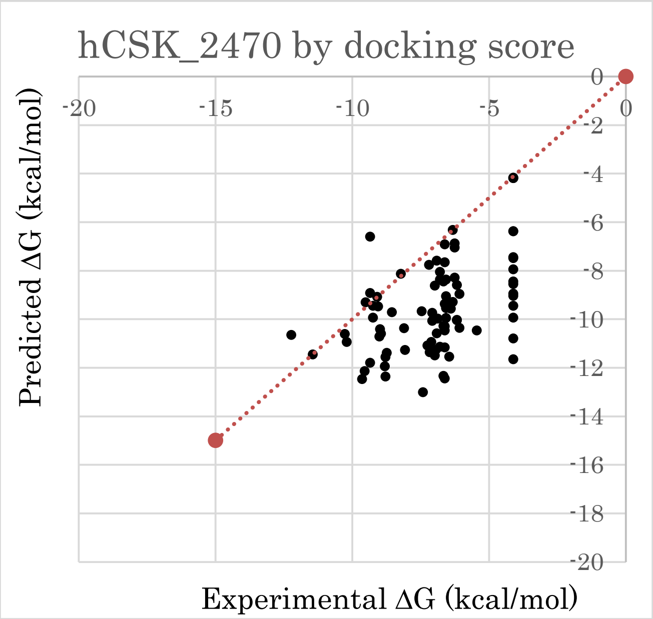
hCSK_2470 by docking score
Predicted ΔG (kcal/mol)
Experimental ΔG (kcal/mol)
-20
-15
-10
-5
0
-2
-4
-6
-8
-10
-12
-14
-16
-18
-20

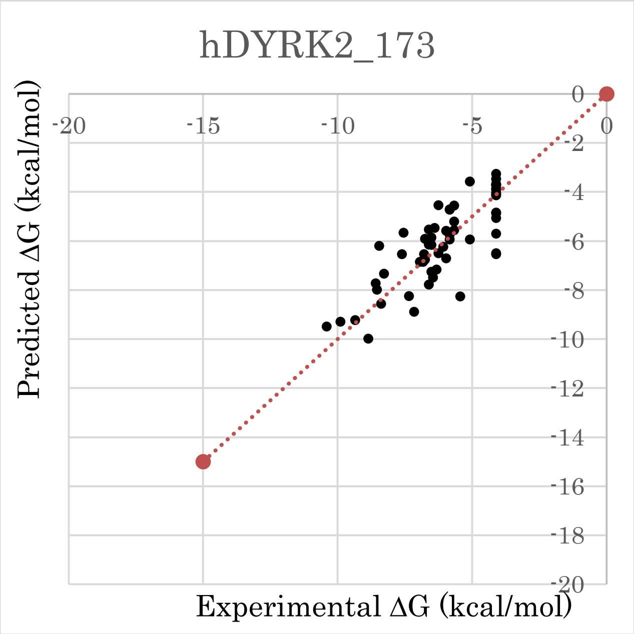
hDYRK2_173
Predicted ΔG (kcal/mol)
Experimental ΔG (kcal/mol)
-20
-15
-10
-5
0
-2
-4
-6
-8
-10
-12
-14
-16
-18
-20

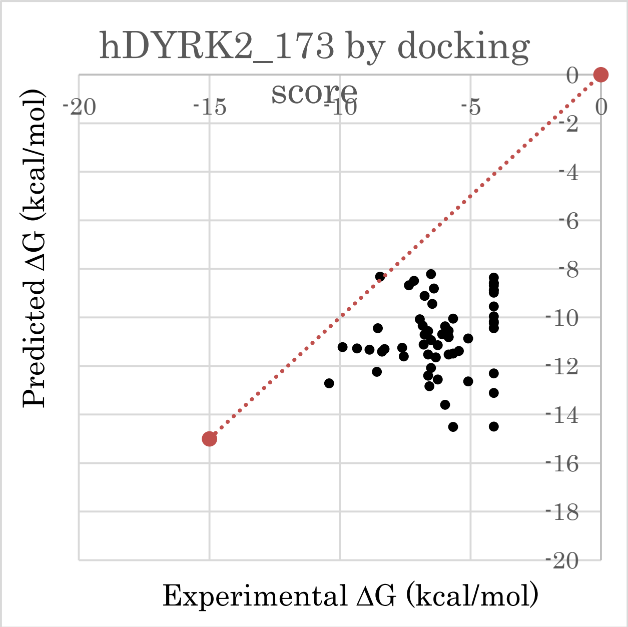
hDYRK2_173 by docking score
Predicted ΔG (kcal/mol)
Experimental ΔG (kcal/mol)
-20
-15
-10
-5
0
-2
-4
-6
-8
-10
-12
-14
-16
-18
-20

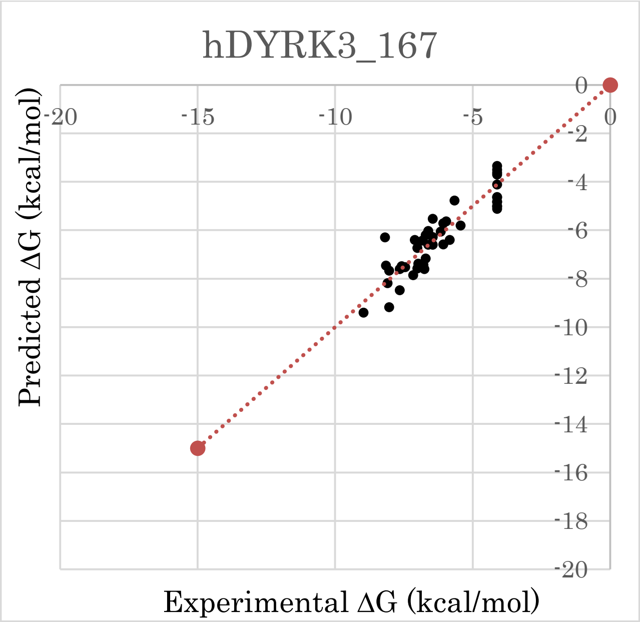
hDYRK3_167
Predicted ΔG (kcal/mol)
Experimental ΔG (kcal/mol)
-20
-15
-10
-5
0
0
-2
-4
-6
-8
-10
-12
-14
-16
-18
-20

Figure S3 Correlation between the calculated and experimental ΔG values. The LOO cross validation results by the docking score QSAR method (λ=0.002, W=20, N=0.5) are in the left column. The naive docking results are in the right column. The blue dotted lines are the y=x lines.

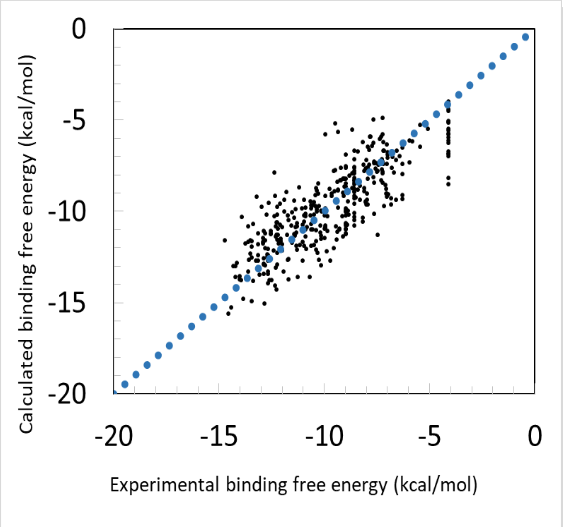

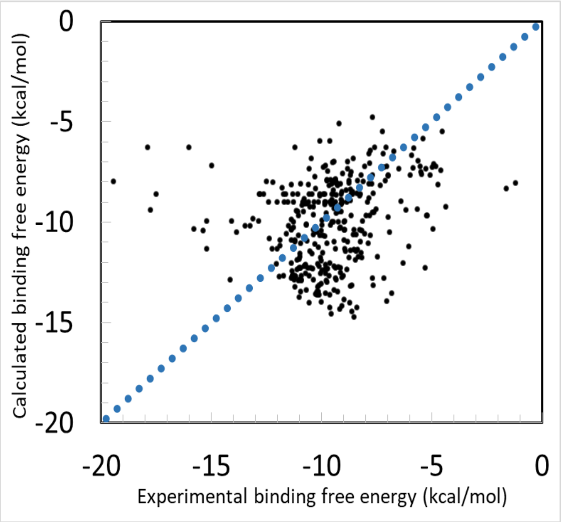

MMP3

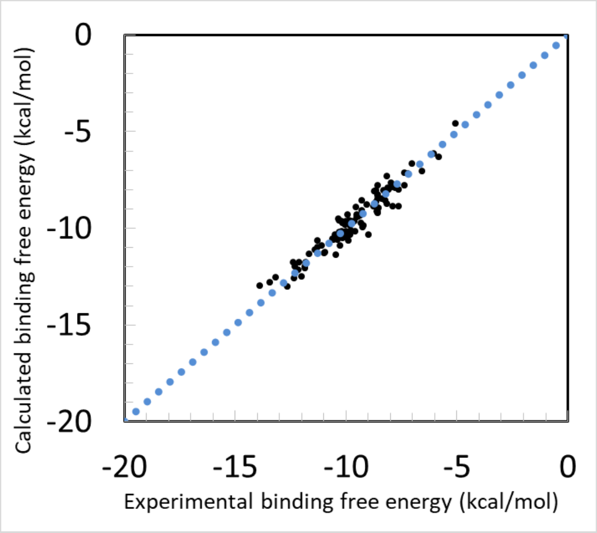

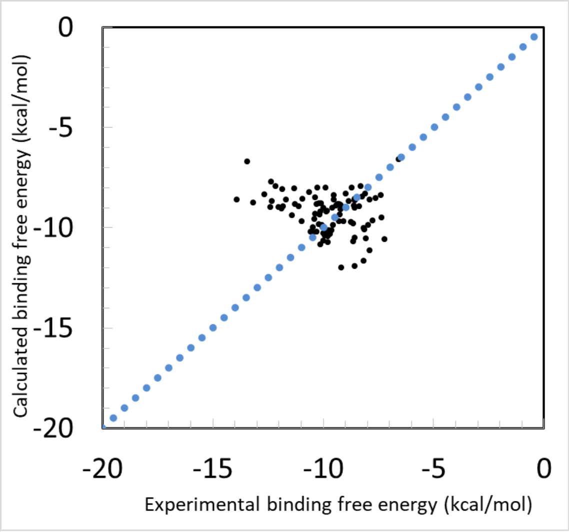

MMP7

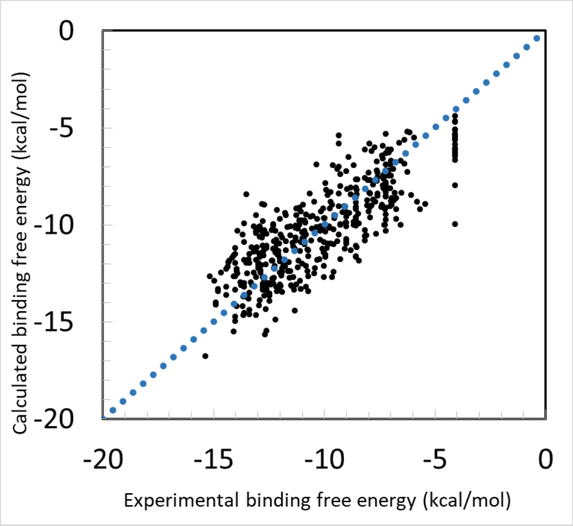

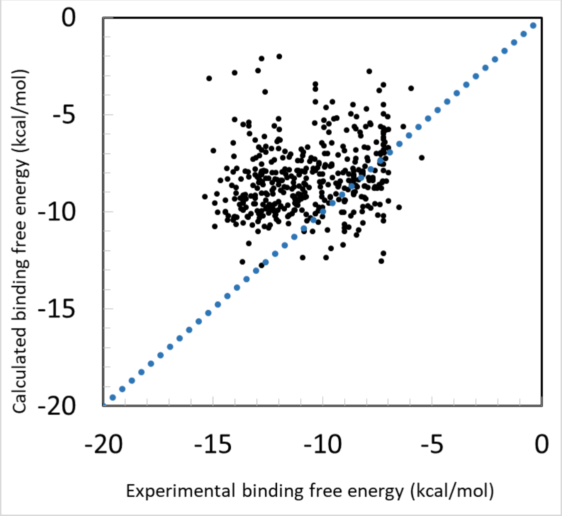

MMP9

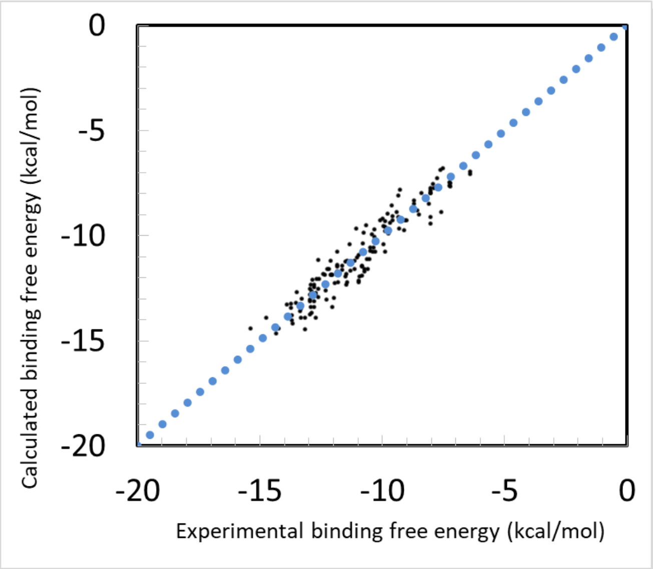
Calculated binding free energy (kcal/mol)
0
-5
-10
-15
-20
-20
-15
-10
-5
0
Experimental binding free energy (kcal/mol)

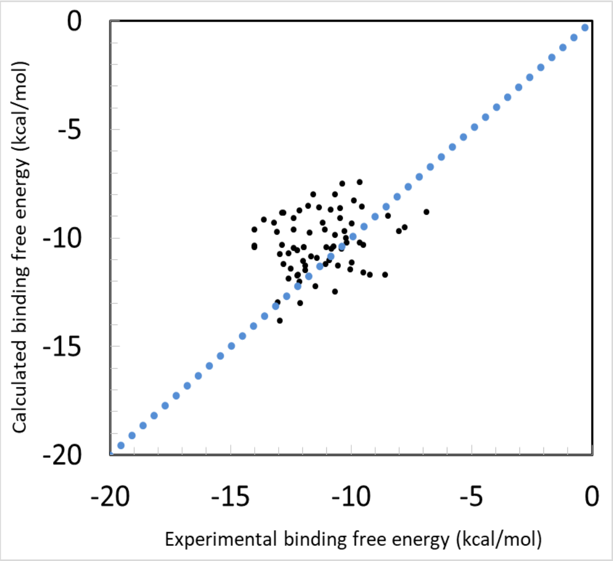
Calculated binding free energy (kcal/mol)
0
-5
-10
-15
-20
-20
-15
-10
-5
0
Experimental binding free energy (kcal/mol)

MMP13
